# A Review on Current Strategies for the Modulation of Thermomechanical, Barrier, and Biodegradation Properties of Poly (Butylene Succinate) (PBS) and Its Random Copolymers

**DOI:** 10.3390/polym14051025

**Published:** 2022-03-03

**Authors:** Mario Iván Peñas, Ricardo Arpad Pérez-Camargo, Rebeca Hernández, Alejandro J. Müller

**Affiliations:** 1 Institute of Polymer Science and Technology ICTP-CSIC, Juan de la Cierva 3, 28006 Madrid, Spain; marioivan.penas@ehu.eus; 2 POLYMAT and Department of Polymers and Advanced Materials: Physics, Chemistry and Technology, Faculty of Chemistry, University of the Basque Country UPV/EHU, Paseo Manuel de Lardizabal 3, 20018 Donostia-San Sebastián, Spain; 3 Beijing National Laboratory for Molecular Sciences, CAS Key Laboratory of Engineering Plastics, Institute of Chemistry, Chinese Academy of Sciences, Beijing 100190, China; riky0712@gmail.com; 4 Ikerbasque, Basque Foundation for Science, Plaza Euskadi 5, 48009 Bilbao, Spain

**Keywords:** poly (butylene succinate), copolymerization, biocomposites, mechanical properties, crystallization, barrier properties, degradation

## Abstract

The impact of plastics on the environment can be mitigated by employing biobased and/or biodegradable materials (i.e., bioplastics) instead of the traditional “commodities”. In this context, poly (butylene succinate) (PBS) emerges as one of the most promising alternatives due to its good mechanical, thermal, and barrier properties, making it suitable for use in a wide range of applications. Still, the PBS has some drawbacks, such as its high crystallinity, which must be overcome to position it as a real and viable alternative to “commodities”. This contribution covers the actual state-of-the-art of the PBS through different sections. The first section reviews the different synthesis routes, providing a complete picture regarding the obtained molecular weights and the greener alternatives. Afterward, we examine how different strategies such as random copolymerization and the incorporation of fillers can effectively modulate PBS properties to satisfy the needs for different applications. The impact of these strategies is evaluated in the crystallization behavior, crystallinity, mechanical and barrier properties, and biodegradation. The biodegradation is carefully analyzed, highlighting the wide variety of methodologies existing in the literature to measure PBS degradation through different routes (hydrolytic, enzymatic, and soil).

## 1. Introduction

Plastic consumption has undergone an incredible increase over the last decades, reaching a record of 367 million tons produced in 2020 worldwide, with a rise of 5.2% with respect to 2017 [1] and a slight decrease of 0.3% compared to 2019 due to the COVID-19 pandemic [2]. Since the 1950s, worldwide plastic production has increased by a factor of more than 200, with a compound annual growth rate (CAGR) of 8.4% [3,4,5]. The forecast exposes a CAGR of 10% for 2018–2023 [6]. As plastic production increases, plastic waste follows a similar increment. This fact is a significant problem, as most plastic materials are single-use products, which implies that they end up in the environment, such as landfills, or directly in the oceans, remaining there for many years. Over the last decades, research on biodegradable polymers has exponentially increased, motivated by the need to diminish human impact on the environment and reduce global plastic pollution, which is also related to the emission of greenhouse gases [7]. Plastic wastes produced within the last seven decades have reached the astonishing amount of 6300 million tons [3,6], setting up plastic pollution in the spotlight as a highly concerning problem to be solved with haste. Among the multiple applications, packaging leads plastic wastes production, with almost 150 million tons in 2015, of which less than 4% were recycled [4]. Additionally, the more concerning fact is that about 1–5% of all plastics end up in marine environments, as around 80% of oceanic wastes come from landfills [8]. The direct consequence of this is the production of microplastics, which cause important damage in the terrestrial and marine wildlife [7], while also affecting human beings, not only producing health issues, but also damaging tourism, fishing, and shipping industries [9]. Although the COVID-19 pandemic has slightly diminished the total production of plastics, the problem of wastes has increased for all the single-use plastic products required in this pandemic situation, such as masks, face shields, gloves, and other personal protective equipments [4]. Within this context, research on biodegradable polymers, defined as those “whose chemical and physical characteristics undergo deterioration and completely degrade when exposed to microorganisms, aerobic and anaerobic processes”, has attracted increasing attention [10].

Poly (butylene succinate) (PBS or PBSu), also known as poly (tetramethylene succinate) (PTMS), is an aliphatic polyester that can be included in fossil-based biodegradable polymers. However, many advances have been made in preparing 100% bio-based PBS (Bio-PBS) produced from bio-based 1,4-butanediol [11]. Among the group of bioplastics, polyhydroxyalkanoates (PHAs), which can be produced in aqueous environments by the actions of different microorganisms [7], present many advantages that make these materials suitable for many applications, such as packaging, biomedical devices, electronics, and agricultural purposes, among others [12]. Notwithstanding the higher production of PBS compared to PHAs (4.3% vs. 1.2% of total bioplastics production in 2019) [13], as well as the higher cost of PHAs against PBS (USD 4000–15,000/t of PHAs vs. USD 2000–5000/t of PBS) [12,14,15], make PBS a more suitable candidate to be used for commercial purposes in the field of biodegradable plastics. Furthermore, a drop in price for Bio-PBS by almost half is expected, a move from USD 4400/t to prices of USD 2700/t [16].

PBS presents interesting physicochemical properties; it is biodegradable and nontoxic, making this polymer a good candidate for various biomedical applications, packaging, agriculture, and others. In an extensive review by Gigli et al., the main biomedical applications of PBS are summarized, including its employment as scaffolds for tissue engineering or matrixes for drug delivery [17]. In agriculture, PBS has been investigated to develop biodegradable polymeric mulch films to reduce plastic accumulation in soil [18]. PBS has also been studied for the fabrication of soft packaging because, in comparison to poly (lactic acid), PBS is more flexible with a higher value of elongation at break, and presents similar good barrier properties to oxygen and water vapor [19]. Despite extensive research on PBS and its potential applications, the widespread employment of this polymer is still limited due to its relatively high cost. Hence, many strategies are being developed to, on the one hand, lower the cost of PBS and, on the other hand, modify the mechanical, physicochemical, and thermal properties of PBS to adjust degradation times and meet specific requirements for selected applications. Such approaches include the formation of blends, the synthesis of copolymers, and the addition of filler materials to prepare composite materials [20]. Over the last few years, many reviews have provided a thorough analysis of the thermal, mechanical, and biodegradation properties of PBS in comparison to other biodegradable polyesters [21], in PBS composites and nanocomposites with incorporated natural fillers [5], and in blends with other polymers, most notably with poly (lactic acid) (PLA) [22]. It is important to remark that, even if the blending of PBS with synthetic and natural polymers constitutes one of the most straightforward routes to modulate PBS properties, PBS-based blends are often immiscible, leading to phase separation and hence poor mechanical properties. One alternative is using compatibilizers or crosslinkers in the blend, aiming to improve phase mixing in the blends. However, such a strategy might compromise the degradability of the material [23]. In this regard, a recent study has shown the employment of a PBS-based copolymer as an effective compatibilizer in a compostable ternary blend made of PBS, random PBS-based copolymers, and plasticized wheat flour [24].

For PBS, random copolymerization constitutes a common strategy for combining the desired properties of two different homopolymers. In PBS copolyesters, the comonomer composition strongly influences the crystallization behavior, which in turn affects their biodegradation, thermal, and mechanical properties [25]. It follows that a thorough investigation of the crystallinity behavior of PBS copolymers as a function of the comonomer composition is necessary to design PBS-based materials with the physical and biodegradation properties required for specific applications. In the current review, we summarize and quantitatively compare the results corresponding to PBS copolyesters synthesized in our group and other research groups to determine, on the one hand, the influence of different synthetic routes on the resulting molecular weight of PBS copolyesters and, on the other hand, the impact of the comonomer content on the crystallization behavior and resulting mechanical properties of the selected PBS copolyesters, including a series of biodegradable PBS-*ran*-PCL synthesized and characterized in our group [26]. Such analysis is extended to PBS composite films, for which the effect of the incorporation of the selected fillers on the mechanical and barrier properties is analyzed. In a recent study from our group, authors presented the peculiar rheological properties for these PBS-*ran*-PCL copolymers, and their applications such as hot melt adhesives [27]. Moreover, a thorough analysis of experimental protocols aimed to determine the biodegradation of PBS and its copolyesters is provided, with emphasis on PBS enzymatic degradation. Finally, future perspectives for the design of PBS-based materials with tailored properties are discussed.

## 2. Synthesis of PBS

Poly (butylene succinate) is obtained from two monomers: succinic acid (SA) and 1,4-butanediol (BD). SA can be obtained through the hydrogenation of fossil-derived maleic acid (anhydride) or 1,4-butanediol. The BD is produced through the hydrogenation of 1,4-butynediol, previously obtained from acetylene and formaldehyde. The BD can also be obtained through the hydrogenation of methyl maleate ester derived from maleic anhydride. In order to move to greener production methods, SA can be prepared through fermentation, whereas it is possible to obtain the BD monomer from a genetically modified organism [28,29]. The polymerization routes for the production of PBS can be divided into two categories: petroleum-based synthesis and bio-based polymerization. The petroleum-based synthesis relies on polycondensation reactions, generating higher molecular weights (MW) than the bio-based polymerization. However, the latter is based on enzymes as catalysts of the reaction, representing a greener alternative. The description of the experimental methodology for each of the polymerization routes is provided below. 

### 2.1. Petroleum-Based Synthesis: Transesterification Polymerization: Melt, Chain Extender, and Solution

**Scheme 1:**
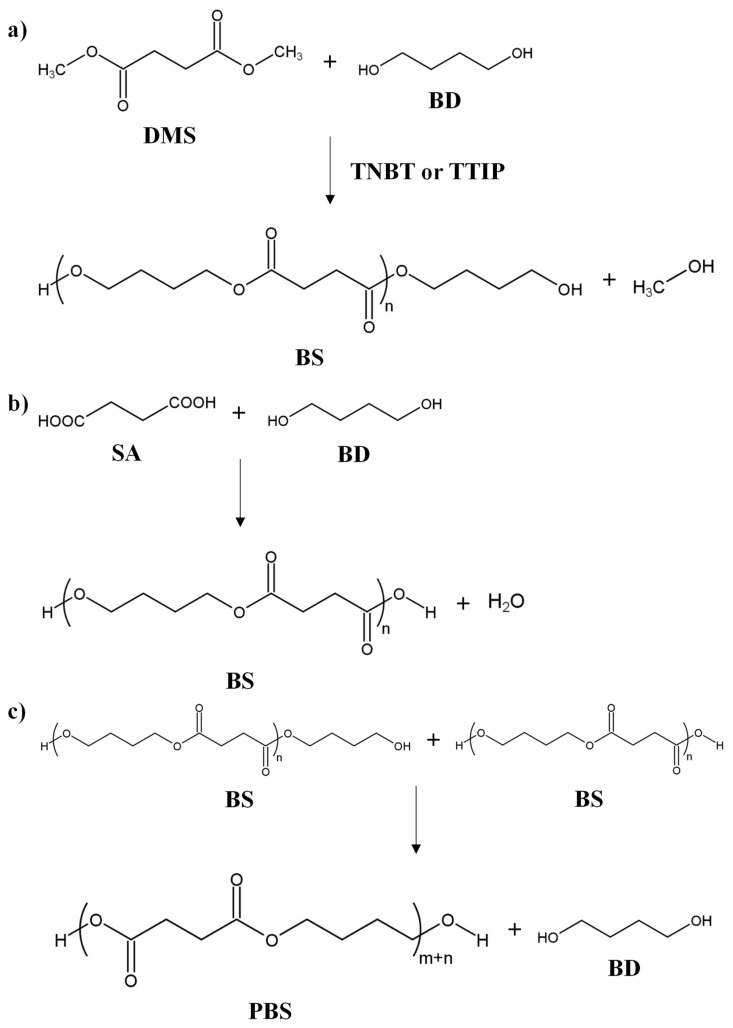
Transesterification polymerization of PBS: (**a**) transesterification step; (**b**) direct polymerization step; (**c**) polycondensation step.

The petroleum-based synthesis is based on transesterification polymerization (shown in Scheme 1), carried out in the melt or in solution. Through these methods, PBS of intermediate and high MWs can be obtained. 

**Figure 1:**
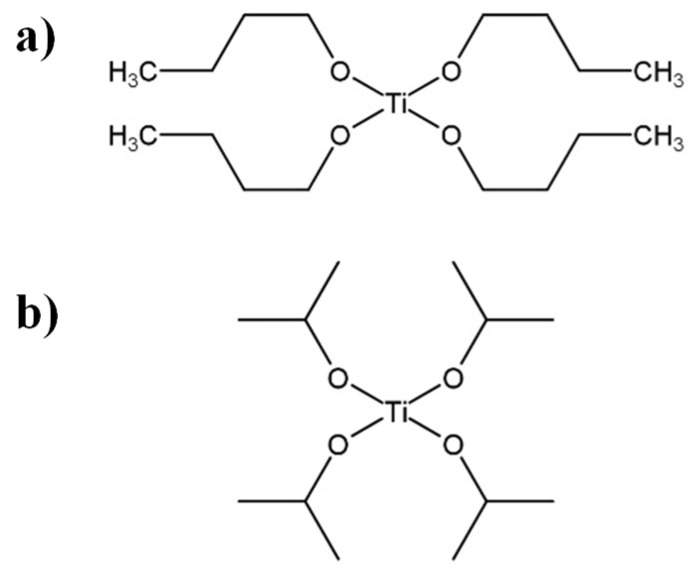
Chemical structures of 9 different catalysts employed in the transesterification step: (**a**) titanium (IV) butoxide (TNBT) and (**b**) titanium (IV) isopropoxide (TTIP).

Intermediate MWs, e.g., the number average molecular weight, *M_n_* ~60,000 g/mol and the weight average molecular weight, *M_w_* ~100,000 g/mol [30,31], are obtained through a transesterification polymerization carried out in the melt. This polymerization employs dimethyl succinate (DMS) and BD, as monomers, in stoichiometric relation or with an excess of BD below 10 mol%. Titanium (IV) butoxide (TNBT) or titanium (IV) isopropoxide (TTIP) are commonly used as catalysts of the reaction [32] (see Figure 1). Before the reaction process, the reactor is filled with nitrogen at room temperature to remove air and avoid oxidation during the transesterification step. After that, the reaction system is heated at a temperature ranging from 150 °C to 190 °C, with constant stirring and under a nitrogen atmosphere to start the transesterification reaction (see Scheme 1a). Then, a distillation step is needed to discard most of the methanol and water produced during the reaction. In a second stage, polycondensation (see Scheme 1c) is carried out under vacuum at a higher temperature to remove the BD formed in the reaction and polymerize the oligomers to the polymer [33].

**Scheme 2:**
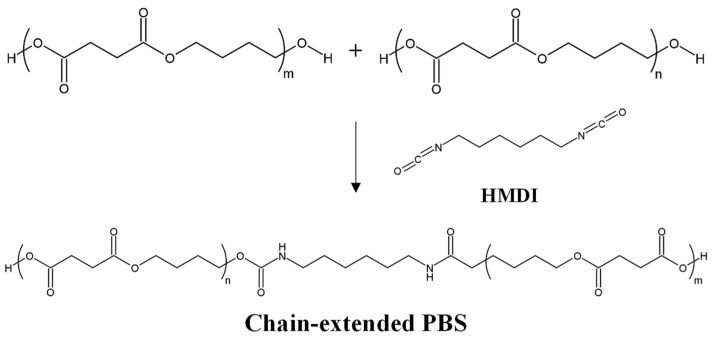
Chain-extension step with hexamethylene diisocyanate (HMDI) to produce high-MW PBS.

A second option for petroleum-based syntheses is the direct polymerization (see Scheme 1b) of both monomeric units (SA and BD), starting from dicarboxylic acids and alkyl diols. High MWs can be obtained by using a chain-extension step. Direct polymerization can be carried out in the melt or in solution. In the melt, the polymerization consists of two steps: first, the esterification reaction occurs at temperatures from 150 °C to 200 °C, under atmospheric pressure, or in a low vacuum. In the second step, the polycondensation is carried out under a high vacuum at a higher temperature (220–240 °C) for deglycolization. It is important to note that both steps should be done under a nitrogen atmosphere to avoid oxidation [34]. For high-MW PBS production, an extra chain-extension step (see Scheme 2) is added to the melt condensation polymerization, achieving *M_n_* ~80,000 g/mol and *M_w_* ~250,000 g/mol. Some authors have reported even higher values: *M_n_* ~180,000 g/mol and *M_w_* ~450,000 g/mol [35], *M_w_* ~500,000 g/mol [36], or up to *M_w_* ~1,000,000 g/mol [37]. These values are supported by Showa Denko, which commercially produced high-MW PBS (Bionolle) with a chain extender (hexamethylene diisocyanate, HMDI), reaching *M_n_* ~200,000 g/mol and *M_w_* ~300,000 g/mol (see Scheme 2). A chain extender with two functional groups can react with the terminal –OH or –COOH of PBS. The reaction conditions for the chain extension are not as critical as the direct melt polycondensation. Therefore, the chain extender incorporation decreases the biosafety and could affect the biodegradability of PBS, which might prevent the employment of the so-obtained PBS in food packaging. Many chain extenders have been investigated, e.g., isocyanate [31], oxazoline [38], anhydride, biscaprolactamate [39], silazane [40], and epoxy compound [36]. Diisocyanate and anhydride are suitable for extending the –OH of the PBS, whereas oxazoline and epoxy compounds are used to extend the –COOH groups of PBS [32].

In the polymerization from solution, the monomers are dissolved in a solvent, such as xylene or decahydronaphthalene. This procedure improves the removal of the small molecular materials formed in the reaction process. Both reactions, esterification, and polycondensation proceed at lower temperatures, which avoids the oxidation of PBS, although the reaction time increases [32]. Regarding the MWs obtained, *M_n_* ~120,000 g/mol and *M_w_* ~280,000 g/mol can be achieved [41], although lower values are also obtained [42,43].

### 2.2. Bio-Based Polymerization: Enzymatic Synthesis

**Scheme 3:**
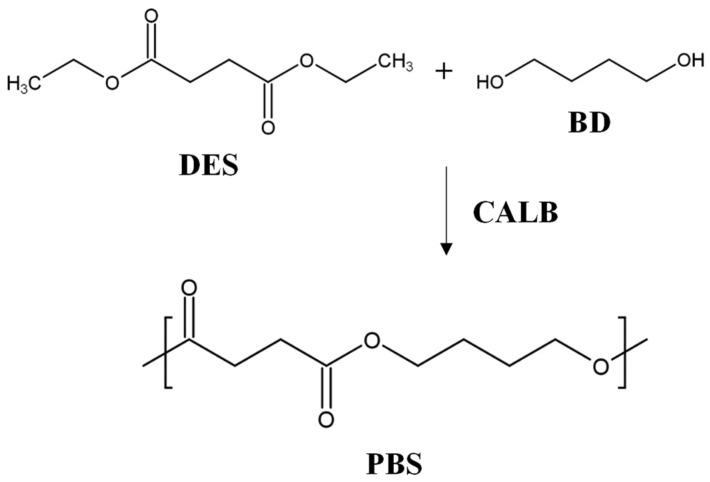
Enzymatic synthesis of PBS by the employment of CALB.

This method is relatively recent with respect to petroleum-based synthesis and presents the advantages of milder reaction conditions and the absence of residual metals and metal salts. *Candida antarctica* lipase B (CALB) is usually employed as a catalyst in synthesizing PBS from the monophasic reaction mixtures of diethyl succinate (DES) and 1,4-butanediol (see Scheme 3). In this case, the reaction temperature dramatically affects the MW of the polymer. In general, reaction temperatures are below 100 °C, with reaction times of 24 h. Another remarkable result obtained from this procedure is the narrow dispersity index of the PBS obtained. However, the MW is low compared to other polymerization methods [44]. Debuissy et al., reported a “green” enzymatic procedure to obtain PBS starting from telechelic hydroxylated poly((r)-3-hydr-oxybutyrate) (PHB-diol) oligomers and employing CALB and BD in a single-step process. The same authors also described another procedure for this enzymatic synthesis, where the main difference is that the PHB-diol oligomers were introduced after 24 h of the CALB-catalyzed reaction [45]. Cyclic butylene succinate oligomers have also been obtained through enzymatic ring-opening polymerization (eROP) employing CALB as the catalyst at temperatures below 100 °C. The obtained oligomers presented low MWs: 4700 g/mol and 6100 g/mol for *M_n_* and *M_w_*, respectively [46]. 

## 3. PBS Copolyesters

This section illustrates the effect of the preparation conditions of selected PBS-based copolyesters on (a) the MW and (b) the general trends found in the literature on the effect of comonomer content on the crystallization behavior and mechanical properties. All the results are plotted as a function of the PBS content. 

### 3.1. Influence of the Preparation Conditions on the Molecular Weight 

**Figure 2:**
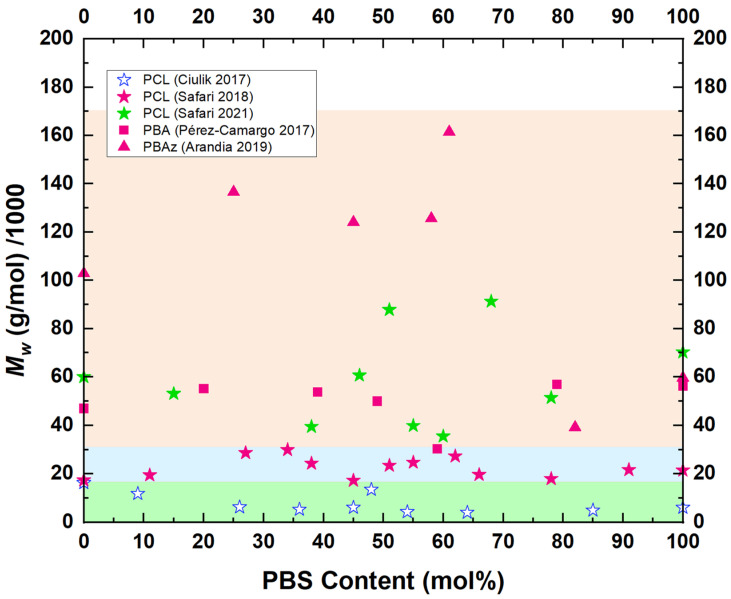
Variation of the weight average molecular weight (M_w_) with PBS composition. The different comonomers are indicated in the legend. The green area corresponds to low-M_w_ copolymers (obtained by enzymatic synthesis), the blue covers intermediate-M_w_ copolymers, and the orange region indicates high-M_w_ copolymers.

PBS copolyesters are obtained through the same experimental procedures, transesterification, or enzymatic synthesis described for the PBS homopolymer in Section 2. Figure 2 shows the variation of the MW with the PBS composition for representative PBS copolymers synthesized previously by some of us and reported in the literature, such as PBS-*ran*-ε-caprolactone (PBS-*ran*-PCL), PBS-*ran*-butylene adipate (PBS-*ran*-PBA), and PBS-*ran*-butylene azelate (PBS-*ran*-PBAz). 

### 3.2. Enzymatic Synthesis for PBS-Based Copolyesters

As can be observed in Figure 2, the use of enzymatic synthesis to prepare PBS-*ran*-PCL copolyesters results in polymers with lower MWs than those obtained for PBS-*ran*-PCL copolyesters synthesized by transesterification synthesis. The experimental procedure followed for the synthesis of PBS-*ran*-PCL through transesterification is similar to that followed for the synthesis of PBS-*ran*-PBA and PBS-*ran*-PBAz copolyesters. 

Different polymerization pathways were followed for obtaining PBS-*ran*-PCL copolyesters, giving rise to a wide range of MWs. For the lowest *M_w_* copolyesters (4000–14,000 g/mol), an enzymatic synthesis was performed, starting from dimethyl succinate and 1,4-butanediol, at a mild mixing temperature (below 100 °C) to obtain cyclic BS oligomers. Later on, the enzyme (immobilized lipase CALB) was added to the reaction medium (in a ratio of 1:1 to the reactants). The cyclization reaction was maintained for 48 h under nitrogen flow. The preparation methodology continued with the dispersion of the reaction mixture in chloroform and the recovery of the enzyme by filtration. The copolyesters were finally obtained by mixing the cyclic reactants (BS and CL) with CALB (50% *w*/*w* relative to the total mass of reactants) at 130 °C for 24 h under a nitrogen flow, and finished with the dispersion in chloroform and recovery of the enzyme, as in the previous step for the obtaining of cyclic BS oligomers [46].

### 3.3. Transesterification/ROP Reaction

Safari et al. synthesized PBS-*ran*-PCL copolyesters with different *M_w_* ranges following a transesterification/ROP reaction and a polycondensation step, mixing dimethyl succinate, 1,4-butanediol, and ε-caprolactone at different ratios. The first reaction was conducted at 160 °C for 4 h, and later on, the polycondensation step was performed at 190 °C at a high vacuum, in the case of the medium *M_w_* copolymers (17,000–30,000 g/mol) [47]. For the high *M_w_* copolyesters (35,000–91,000 g/mol), the polycondensation step lasted longer (6 h instead of 4 h) and was carried out at higher temperatures (220 °C instead of 190 °C) [26].

PBS-*ran*-PBA copolyesters were synthesized following a two-stage melt polycondensation reaction (polycondensation and postpolycondensation) starting from a bio-based SA (obtained by fermentation of glucose), BD, and adipic acid (AA). For the esterification step, the reaction temperature was kept at 200 °C. Later on, TTIP catalyst was introduced into the reactor (210 °C, with argon flux). For the second step (postpolycondensation), the temperature was slowly raised to 230 °C, and reduced pressure was employed to avoid uncontrolled foaming and to minimize oligomer evaporation. This procedure led to copolymers with *M_w_* in the range 30,000–57,000 g/mol and a polydispersity index of 1.6–2.0, as observed in Figure 2 [48]. Similarly, a two-stage melt polycondensation has been employed to synthesize PBS-*ran*-PBAz copolyesters with some modifications: esterification of dimethylazelate (DMAz), SA, and BD was carried out at 200 °C at atmospheric pressure employing TNBT as the catalyst. The polycondensation step was carried out at reduced pressure, and for this step, the temperature was raised to 250 °C. The MWs of the so-obtained copolymers were in the 40,000–160,000 g/mol (*M_w_*) range, improving the latter in the case of the copolymers in comparison with the homopolymers [49]. Other authors have followed a similar procedure to synthesize PBS-*ran*-PBAz copolyesters, giving rise to lower *M_w_* than the one observed in Figure 2, due to milder synthesis conditions [50].

### 3.4. Crystallization Behavior in PBS Copolyesters as Determined by DSC 

PBS is a promising material since it combines good thermal and mechanical properties with easy processability and relatively low cost. PBS possesses a relatively high melting temperature (110 °C) and a low glass transition temperature (−25 °C) crucial for applications. Its mechanical properties are comparable to commodity nonbiodegradable polymers, like polyethylene (PE) and polypropylene (PP). PBS is fully biodegradable, but its high crystallinity slows down its degradation rate and induces low barrier properties. Thus, to overcome these disadvantages and tailor the final properties of the PBS, its chain structure is modified, mainly to decrease its crystallinity, either by adding additives or a second phase, e.g., copolymerization. In this way, the range of applications of the PBS-based materials is broadened, and its price is reduced [51,52,53]. 

Different strategies have been used to tailor the properties of PBS, such as blending with other polymers, incorporating natural fibers, nanofillers, or through copolymerization, as block and random copolymers. Here, for brevity, we will focus on the thermal behavior of the PBS-based random copolymers.

**Figure 3:**
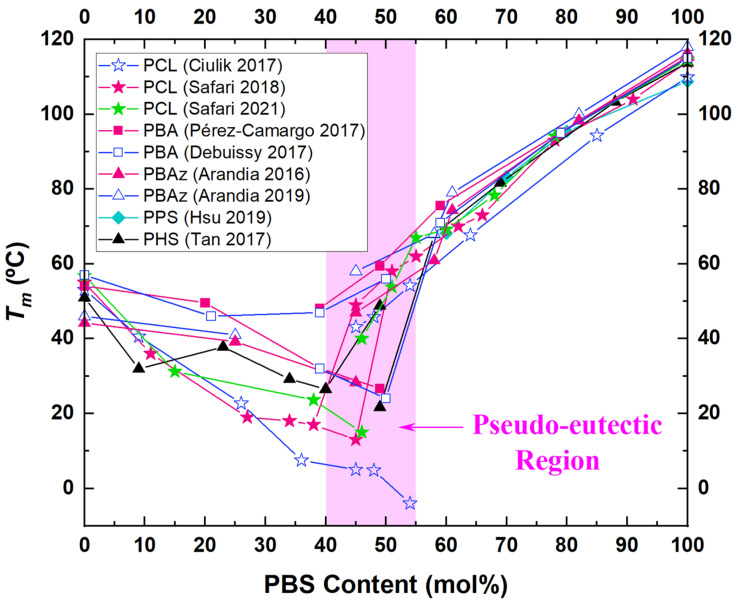
Variation of the melting temperature as a function of the PBS composition for selected copolyesters. Pseudo-eutectic points can be observed in the shaded region.

In general, the random copolymerization allows achieving final properties in between those of the parent components. From a crystallization point of view, different crystallization modalities, namely (a) comonomer exclusion, (b) isomorphism, and (c) isodimorphism, have been reported and recently reviewed [54]. Further details of the different crystallization modes can be found elsewhere [54,55]. Briefly, if we consider a PA-*ran*-PB copolymer, three scenarios can occur: (a) Total Comonomer Exclusion: In the PA-rich phase, B comonomers are totally excluded from the PA crystals, and vice versa. Such excluded B or A comonomers will hinder the crystallization of the PA- or PB-rich crystals, respectively. Therefore, only the PA- and PB-rich phases can crystallize with low B or A comonomer content, e.g., 20% of the randomly distributed co-units along the chain are enough to inhibit the crystallization completely [54]; (b) Isomorphism: The PA-*ran*-PB copolymer can crystallize in a single phase due to the total inclusion (cocrystallization) of the respective comonomer (isomorphism); (c) Isodimorphism: In between cases (a) and (b), the PA-rich phase allows a partial inclusion of B comonomers and vice versa, making possible the crystallization in all the composition range. For isodimorphic random copolymers, when the melting point (*T_m_*) is plotted as a function of the comonomer content, as in Figure 3, a clear pseudo-eutectic behavior is typically obtained. Considering Figure 3 as an example, to the left of the pseudo-eutectic point or region, the PA-rich phase crystallizes with some inclusion of B comonomers; thus, structurally, the copolymer crystallizes with a PA unit cell with distortions, i.e., expansion or shrinkage of the unit cell, caused by the inclusion of B comonomers in the crystal lattice. The opposite, i.e., the PB unit cell with A comonomers inclusion, occurs at the right side of the pseudo-eutectic point or region. In some cases, it has been demonstrated that both PA- and PB-rich phases can be formed at the pseudo-eutectic point [26,46,47,48,51,54,56,57,58,59,60,61].

Figure 3 shows that, typically, the PBS-based copolymers display an isodimorphic behavior, exhibiting a clear pseudo-eutectic point. The determination of the position of the pseudo-eutectic point is still unclear; however, it seems that it depends on the crystallization ability of the material [62,63,64]. The PBS possesses a symmetrical chemical structure, adopting an all-*trans* conformation within its unit cell, facilitating the formation of a highly ordered crystalline structure [63]. This provides a high melting point to the PBS and a more stabilized crystalline structure than other copolyesters. In general, those polymers that can form isodimorphic copolymers with PBS are characterized by a strong crystallization ability [63]. Thus, the PBS-based copolymers display a pseudo-eutectic point or region around 50%, as the PBS-*ran*-PBAz [57,58,59,60], PBS-*ran*-PBA [48,51], and PBS-*ran*-PCL [26,46,47,56,61] studied by our group and shown in Figure 3. In these random copolymers, the pseudo-eutectic point or region displays the crystallization of both crystalline phases.

Pérez-Camargo et al. [48,51,54] found that for 50:50 PBS-*ran*-PBA copolymers, the “double-crystallization” behavior depends on the cooling rate. Similar results are also reported by Arandia et al. [57,58,59,60] in 58:42 PBS-*ran*-PBAz copolymers and Safari et al. [26,46,47,56,61] in 45:55 PBS-*ran*-PCL copolymers of different MWs. In these systems, a slow cooling rate, e.g., 5 °C/min, is favored for the crystallization of the PBS-rich phase, which has ample time (note that the crystallization of the PBS occurs first) to develop spherulites with a relatively high degree of crystallinity. This forces the second component, i.e., PBA, PBAz, or PCL, to crystallize in the confined interlamellar spaces of the PBS-rich crystalline lamellae. However, such confinement effect at slow cooling rates hinders the crystallization of the second phase.

In contrast, at faster cooling rates, e.g., 50 °C/min, the PBS-rich phase in 58:42 PBS-*ran*-PBAz crystallizes during cooling but develops a lower degree of crystallinity. This gives a chance to the PBAz to crystallize during the fast cooling within the interlamellar regions of the PBS spherulitic templates [57]. For 50:50 PBS-*ran*-PBA and 45:55 PBS-*ran*-PCL copolymers, the rapid cooling inhibits the crystallization during the cooling of the PBS, allowing the PBA- or PCL-rich phases to develop crystallinity. In the subsequent heating, both crystalline phases displayed a sequential cold-crystallization and melting. The PBS crystallizes and melts at higher temperatures than the other phase, either PBA or PCL; for more details, see [48,54]. In the 40:60 PBS-*ran*-PBA, it was found that only favorable thermodynamic conditions, e.g., slow cooling rates, allow the crystallization of the minority PBS phase. In contrast, the PBA phase is the only one able to crystallize without these conditions [48,65]. Similarly, under isothermal tests, a low crystallization temperature, *T_c_*, favored the formation of both PBA- and PBS-rich phases, while at high *T_c_* a PBS-rich phase was the only one formed. Safari et al. reported similar behavior in 45:55 PBS-*ran*-PCL copolymers. It is important to remark that Safari et al. [26] studied the PBS-*ran*-PCL copolymers in a wide range of MWs, obtaining a pseudo-eutectic behavior in all the cases, and similar trends due to the MWs are above the critical MW for entanglements. In a more recent contribution, Pérez-Camargo et al. [51] found that comonomer inclusion depends on the crystallization conditions. The fast crystallization, i.e., nonisothermal test, favored the BA inclusion inside the PBS crystals, whereas slow crystallization (i.e., isothermal test) strongly limits it. Intermediate crystallization, i.e., a combination of nonisothermal and isothermal tests, generated in a successive self-nucleation and annealing test, causes an intermediate situation. 

Figure 3 shows that most of the PBS-based copolymers possess similar behaviors; however, there are exceptions, e.g., comonomer exclusion and isomorphism, and below, these cases are briefly described.

### 3.5. Pseudo-Eutectic Point at Different Content, Comonomer Exclusion, and Isomorphism in PBS-Based Copolymers

The pseudo-eutectic point at a PBS content of around 50% for PBS-based copolymers has its exceptions, as found by Yu et al. [63]. These authors studied PBS-*ran*-*cis*-butene succinate, PBS-*ran*-PcBS, copolymers with a pseudo-eutectic point located at 20% of PBS content. This means that the PBS mainly dominates the copolymer crystallization. This behavior was surprising since the PBS and the PcBS have the same chain length and similar monomer size, but the PcBS possesses one *cis* double bond in the alkyl unit, generating such a particular behavior. The authors attributed the “atypical” pseudo-eutectic behavior for PBS-based copolymers to the different crystallization abilities. The *cis*-isomer in the PcBS introduces “kinks” into the main chain. This difficulty in forming crystals as perfect as the PBS (i.e., all-*trans* configuration, which generated a more stabilized crystalline structure) resulted in a weaker crystallization ability. 

In the PBS-*ran*-propylene succinate (PBS-*ran*-PPS) copolymers, prepared by Papageorgiou and Bikiaris [66], and more recently by Debuissy et al. [67], the very slow crystallization kinetics of the PPS limited the copolymer crystallization to the PBS-rich phase. Such very slow crystallization is caused by the odd chain length of the 1,3-propanediol group [68]. For the as-received samples directly obtained from the synthesis, the WAXS experiments at RT reveal that the PBS-*ran*-PPS copolymers display PBS unit cells even when it is in the minority phase (i.e., 40%). In contrast, the PPS-rich unit cell is only present with a PPS content from 80% to 90% [66,69]. The behavior dramatically changes when the initial thermal history is erased, revealing a more complex trend. After erasing the thermal history and cooling the sample from the melt, only the PBS-rich compositions can crystallize, while the PPS-rich compositions cannot crystalize, as shown in Figure 3. After crystallization from the melt, such behavior is similar to a comonomer exclusion case. Similar findings were reported by Papageorgiou and Bikiaris [66].

The isomorphic behavior, which is not commonly reported, has been found in PBS-based copolymers. The PBS-*ran*-butylene fumarate (PBS-*ran*-PBF) copolymers [70] can form isomorphic copolymers. Ye et al. [70] found that *T_m_* increased linearly with the PBF content, the melting enthalpies hardly changed, and all the copolymers displayed similar crystal structures. These authors attributed the isomorphism to the match of all-*trans* conformation adopted by PBS and PBF comonomers. Similar behavior was also found by Zheng et al. [71] in multiblock copolymers of PBS-*co*-PBF. In the following contribution, Ye et al. found that in random terpolyester PBS-*ran*-PBF-*ran*-PBA [72], the PBS and the PBF can cocrystallize in an isomorphic mode, despite the presence of the PBA comonomer. Such isomorphism was even found recently in PBS/PBF blends [73]. In this case, the isomorphism is located in the PBS-rich blends due to the strong hydrogen bonding ability of fumarate units [73].

### 3.6. Influence of the Comonomer Content in the Crystallinity

**Figure 4:**
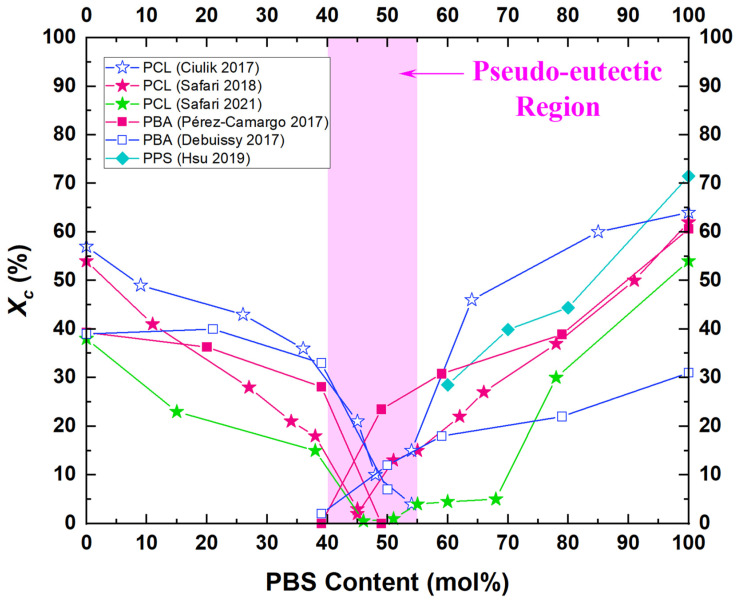
Variation of the degree of crystallinity as a function of the PBS composition for selected copolyesters. Pseudo-eutectic points can be observed in the shaded region.

Figure 4 plots the degree of crystallinity (*X_c_*) as a function of the comonomer content. *X_c_* is calculated according to Equation (1), which is applicable in copolymers.

(1) $$X_{c}=\frac{{\Delta H}_{m}}{{\Delta H}_{m}^{0}\cdot \emptyset }$$

$\Delta H_{m}$ and $\Delta H_{m}^{0}$ are the melting enthalpy and the equilibrium melting enthalpy of the phase under consideration and ∅ is the weight fraction of the phase under consideration. In general, the extent of *X_c_* depression is related to the easiness of comonomer cocrystallization. As Müller et al. [54] pointed out, the decrease of the *X_c_* with the comonomer inclusion indicates that the co-units represent defects for the crystal. For instance, for comonomer exclusion, a strong depression of *X_c_* with the composition is expected since the excluded comonomer decreases the length of the crystallizable sequence (by limiting the number of second comonomer units) included in the crystalline lattice. In contrast, for the isomorphic copolymer, it is reported that *X_c_* remains unchanged or even increases with comonomer content [70], as the comonomer does not represent an interruption for the crystallizable sequences. In the case of isodimorphic copolymers, as shown Figure 4, a pseudo-eutectic behavior of the *X_c_* vs. comonomer content is often obtained due to the partial inclusion of comonomer units. 

As a summary, Figure 3 and Figure 4 show that in PBS-based copolymers, the second phase (i.e., second comonomer) tailors the temperature of the use of the copolymer as well as the crystallinity. Such behavior influences these copolymers’ mechanical properties and degradation, as shown in the following sections.

### 3.7. Effect of Comonomer Content on Mechanical Properties

PBS presents a lower Young’s modulus value compared to other common polyesters, such as polyethylene terephthalate (PET) or polylactic acid (PLA), and similar values for elongation at break to low-density polyethylene (LDPE), polypropylene (PP), or polycaprolactone (PCL) [29]. The mechanical properties range of values commonly reported, for PBS in the literature is: Young’s modulus of 0.5–1 GPa [74,75,76,77], a tensile strength of 30–50 MPa, and elongations at break varying from values below 5% up to 500% [78,79,80,81,82,83,84,85]. The mechanical properties—MW dependence has also been studied in the literature. It has been found that at higher MWs, the elongation at break increases, which ranges from 355% (high MW) to 25.2% (low MW). Similarly, the tensile strength slightly increases with the MW [32].

**Figure 5:**
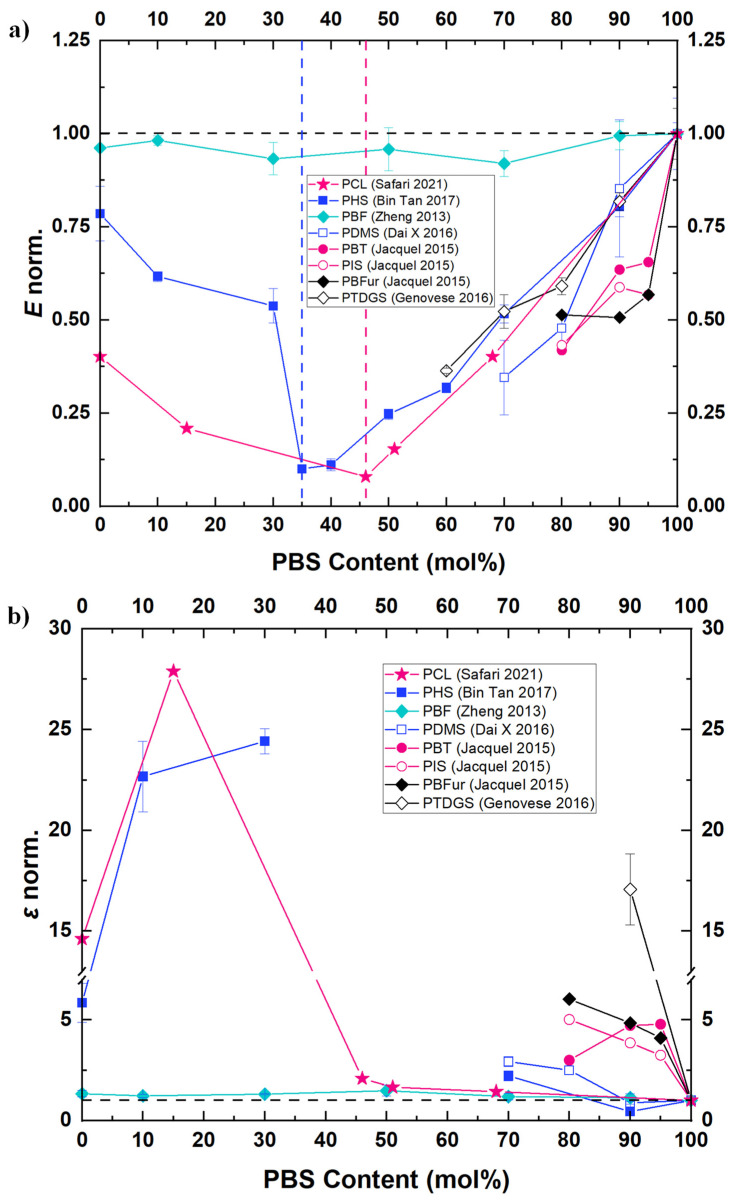
(**a**) Variation of the normalized elastic moduli (*E*) with PBS composition for different copolymers and (**b**) variation of the normalized elongation at break (*ε*) with PBS composition for different copolymers. For a better comparison, the values have been normalized to those of neat PBS. Dashed colored vertical lines reflect a minimum in the elastic moduli, in agreement with the composition of the pseudo-eutectic point observed in the *T_m_* vs. the composition graph (Figure 3).

Figure 5a,b show, respectively, Young´s modulus (*E*) and the elongation at break (ε) obtained from the stress–strain curves of selected PBS-based copolymers plotted against the PBS content of the copolymers. 

Generally speaking, for the PBS homopolymer, introducing a comonomer results in a decrease in the Young´s modulus and an increase in the elongation at break values. Going into detail on the results for each particular copolymer shown in Figure 5a, the variation of the Young’s modulus with PBS content has been determined for the whole range of BS compositions for PBS-*ran*-PCL and PBS-*ran*-PHS copolyesters. For these two copolymers, the variation of *E* is clearly dependent on the composition range, exhibiting a pseudo-eutectic behavior, registering the minimum properties at the pseudo-eutectic point, i.e., at 45% and 35 mol % PBS for PBS-*ran*-PCL [26] and PBS-*ran*-PHS [52], respectively. This is in line with the variation of the *T_m_* and *X_c_* values as a function of the PBS content (Figure 3 and Figure 4, respectively). As expected, these results show a direct correlation of the mechanical properties on *X_c_*. The PBS-*co*-PBF multiblock copolymers show a different trend, since the *E* values remain practically unchanged with the PBS content. This particular trend is caused by the isomorphic-like behavior reported for the PBS-*co*-PBF system. Moreover, the PBS and PBF have similar elastic moduli values [71].

For the other copolymers, shown in Figure 5a, PBS-*ran*-decamethylene succinate (PBS-*ran*-PDMSu) [86], PBS-*ran*-butylene terephthalate (PBS-*ran*-PBT), PBS-*ran*-isosorbide succinate (PBS-*ran*-PIS) and PBS-*ran*-butylene furanoate (PBS-*ran*-PBFur) [87], and PBS-*ran*-thiodiethylene glycol succinate (PBS-*ran*-PTDGS) [88], the variation of *E* with the PBS composition has only been determined for the BS-rich composition range (at compositions higher than ~60 mol% PBS) and show a linear decrease of *E* with the introduction of the comonomer in the PBS-rich compositions. A corresponding increase in the values of the elongation at break is depicted in Figure 5b for these same copolymers. It is important to note that, in the case of PBS-*ran*-PTDGS, the elongation at break increases up to 35 times at 80 mol% PBS with respect to the PBS homopolymer. Such a result is promising for using this copolyester in soft packaging applications [88].

## 4. PBS Nanocomposites: Modulation of Mechanical Properties

**Figure 6:**
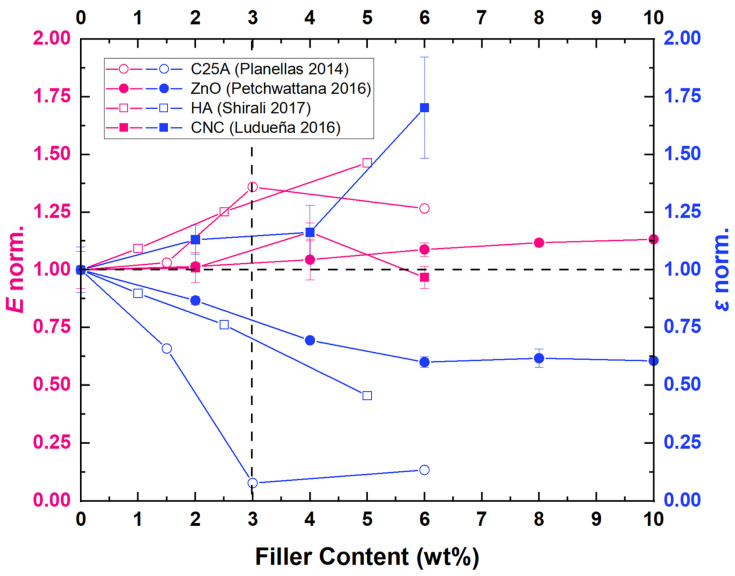
Variation of the elastic moduli (*E*) and elongation at break (*ε*) with filler content. For a better comparison, both parameters have been normalized to that of neat PBS. The dashed black line corresponds to a “critical” filler content.

The incorporation of organic and inorganic fillers to produce nanocomposites are common routes for the modulation of mechanical properties and directly impacts the barrier properties of PBS. Figure 6 shows representative results corresponding to the variation of the mechanical properties, elastic moduli, and elongation at break of PBS nanocomposites as a function of filler content. Here, we have selected PBS/nanoclays nanocomposites, which incorporate organo-modified montmorillonite (CLOISITE^®^ 25A, C25A) and other examples of PBS nanocomposites, such as those incorporating zinc oxide nanoparticles (ZnO), hydroxyapatite nanoparticles (HA), or cellulose nanocrystals (CNC). 

As shown in Figure 6, PBS/nanoclays nanocomposites such PBS/C25A increased their elastic moduli up to 1.25 times at 3 wt% nanofiller. Then, above this concentration, a decrease of the elastic moduli is observed, ascribed to the clay degradation during processing [82]. Several reviews on the development of PBS nanocomposites, through the incorporation of clays and nanoclays as fillers, show different preparation routes, including in situ intercalation, solution casting, melt intercalation, transesterification, and master batch [89]. The types of clays commonly employed for the formation of PBS nanocomposites are mainly constituted by the family of 2:1 phyllosilicates: montmorillonite (MMT), saponite (SAP), and fluorohectorite (FHT) [90]. 

Figure 6 also shows the results corresponding to PBS nanocomposites prepared by incorporating inorganic nanoparticles, like zinc oxide (ZnO) and hydroxyapatite (HA). Incorporating ZnO nanoparticles (100 nm average size) gives rise to a slight increase in the elastic moduli for PBS nanocomposite films up to a ZnO concentration of 10 wt% and a decrease of the values corresponding to the elongation at break. The rigidity of the ZnO nanoparticles, the restriction of the chain entanglement, and the rearrangement of crystallized PBS chains induced by the nanoparticles explained the results found for these PBS nanocomposites [79]. The incorporation of hydroxyapatite nanoparticles also increases the values of the elastic moduli up to 1.5 times at an HA concentration of 5 wt% [91]. 

Finally, the incorporation of bionanofillers constitutes an important field of research nowadays due to the possibility of preparing fully biodegradable polymer nanocomposites. As an example, Figure 6 shows the variation of the mechanical properties for PBS ternary nanocomposites obtained through the mixing of PBS, poly(ethylene glycol) (PEG), and cellulose nanocrystals (CNC) in a weight ratio of 80:20 in PBS:PEG, and increasing amounts of CNC (2, 4 and 6 wt%). PBS/PEG/CNC nanocomposites showed a slight increase of the elastic moduli up to CNC contents of 4 wt%. Above this content, the elastic moduli decreased compared to the pristine PBS because of the poor dispersion of the CNC within the PBS matrix [81]. Other bionanofillers that have been employed to modify the mechanical properties of PBS are isora nanofibers (INF) extracted from *Helicteres isora* by thermo-mechano-chemical treatments [92], wood flour (WF) [93], oil palm mesocarp fibers (OPMF) [65], or konjac fly powders (KFP) [94], among others. 

In summary, the general trend found for PBS nanocomposites is that incorporating nanofillers, even at a low content (less than 6 wt% filler), results in an increase of the elastic moduli and a decrease of the elongation a break. However, many factors play a key role, such as the degree of dispersion, the crystallinity or the orientation of the nanoparticles, and the interactions between the polymer matrix and the nanoparticles, which are necessary to propagate the stress through the nanocomposite. That is why each particular PBS nanocomposite has to be studied separately to extract proper conclusions on the variation of the mechanical properties with the nanofiller content.

## 5. Barrier Properties

The study of barrier properties in the materials field is an important step for their evaluation for food packaging applications and food-related categories [95,96]. It is also important for other applications such as coatings for many different substrates [97] or mulching films that can be employed to protect crops in agriculture [20,98,99]. This type of study must consider both gas and liquid barrier properties, as packages should protect the food from both external gases and liquids. Moreover, the shelf life of the product is also affected by these permeants, water vapor being one of the most relevant [100]. Regarding the liquid barrier properties, the most commonly studied one is water [101,102], although other liquids have also been studied, such as acetic acid, ethanol, or isooctane, among others [103]. 

Nevertheless, having a material that accomplishes the required gas barrier properties is a must, as food needs to be preserved under certain atmospheric conditions. Thus, novel materials should be designed to fulfill all the requirements for food packaging in terms of gas permeability. For this purpose, the most abundant gases in the atmosphere are commonly studied (e.g., water vapor, oxygen, carbon dioxide, and nitrogen) [104]. Some reports have also included helium, argon, methane or hydrogen, or vapors such as methanol or dimethyl carbonate [105,106]. It is crucial to have the oxygen levels under control, as high values could accelerate the enzymatic degradation of the food, whereas too low levels could lead to tissue deterioration. Carbon dioxide is related to antimicrobial properties, whereas nitrogen is used to complete the inside atmosphere of the package, being inert to food and protecting the film from breaking [107].

The most studied and commonly used materials for food packaging are synthetic polymers (e.g., PET, PP, and PE), although PLA has already become one of the most important alternatives to these “commodities” among biodegradable polymers [108,109]. Nonetheless, PBS is promising due to its good processing conditions and its wide range of thermomechanical properties. It also shows similar/enhanced barrier properties compared to PLA, positioning PBS as a real alternative in the field of biodegradable polymeric packages.

**Figure 7:**
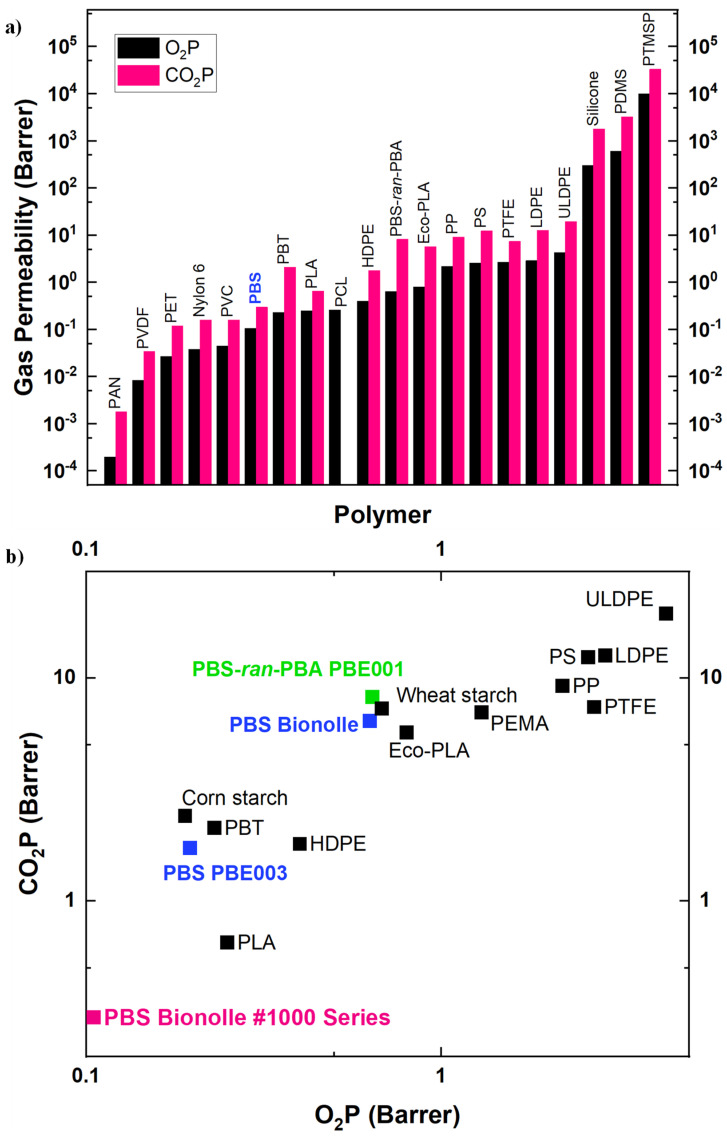
Gas barrier properties (O_2_P and CO_2_P) for several commercial polymers and biopolymers: (**a**) bar chart for a general overview and (**b**) plot for intermediate permeabilities (PBS region). Note that both axes are presented in decimal logarithm form. Data taken from [97,101,103,105,106,109,112,113,114,115,116,117,118,119,120,121,122].

Figure 7a shows an overview of the gas barrier properties (O_2_ and CO_2_) corresponding to several polymers. As it can be appreciated, gas permeabilities of polymeric materials cover a wide range of values, starting from the extremely low values of polyacrylonitrile (PAN) or a liquid crystal polymer (LCP) from Vectra, which present triple bonds or aromatic rings in their structures, to the very high values for silicones, such as polydimethylsiloxane (PDMS) or poly(1-trimethylsilyl-1-propyne) (PTMSP). When expressing the permeabilities in Barrers (1 Barrer = 10^−10^ × ${\mathrm{cm}}_{\mathrm{STP}}^{3}$ × cm × cm^−2^ × s^−1^ × cm × Hg^−1^, where ${\mathrm{cm}}_{\mathrm{STP}}^{3}$ stands for standard cubic centimeter, representing the number of moles of gas that would occupy 1 cm^3^ at standard temperature and pressure), a common unit used to express gas permeability, a range over 10 orders of magnitude can be achieved. In the previous expression, ${\mathrm{cm}}_{\mathrm{STP}}^{3}$ × cm^−2^ × s^−1^ denotes the gas flux through the material, cm comes from the thickness of the film, and cm × Hg^−1^ stands for pressure drop across the material.

Within the group of “commodities” (nonbiodegradable polymers) commonly employed in food packaging, or in other applications where gas permeabilities play an important role (e.g., mulching films for crops protection), we can see that some of them present excellent barrier properties (PVDC), good barrier properties (PET, PVC, and Nylon 6), or average barrier properties (HDPE, LDPE, and PP). In the field of biodegradable polymers, few polymers show excellent barrier properties: chitosan and ethylene vinyl alcohol (EVOH) [95] are two examples. Within the group of biopolymers with good barrier properties, we can find PLA, PCL, PHB, PHBV, or collagen [110], among others. Regarding PBS, it shows slightly enhanced barrier properties compared to those mentioned above, which leaves PBS in an advantaged place towards barrier properties within biodegradable polymers, and similar barrier properties to those of PVA [101,111] (see Figure 7b).

### 5.1. PBS-Based Copolymers

One possibility of modulation of PBS barrier properties is the copolymerization of PBS with different comonomers. A comparison of the permeability of PBS, PBS-*ran*-PBA, and PLA to oxygen and carbon dioxide showed a higher permeability of the three polymers to CO_2_ compared to oxygen, as can be observed in Figure 7a. Among these three polyesters, PBS-*ran*-PBA showed improved barrier properties to both gases with respect to PLA, while PBS showed a lower permeability for oxygen but higher to CO_2_. These results are quite promising for PBS, turning it into an alternative to PLA, one of the most employed polymers for biodegradable packaging films [103]. Other authors showed that PLA/PBS-*ran*-PBA [123] and PLA/PBS [122] (80/20) multilayer blend films achieved similar barrier properties to those of PLA and PBS, and much better than neat PBS-*ran*-PBA.

Genovese et al. studied the influence in oxygen and carbon dioxide permeabilities (i.e., O_2_P and CO_2_P) of different poly(butylene succinate-*ran*-thiodiethylene glycol succinate) (PBS-*ran*-PTDGS) copolymers. It was found that the barrier properties to both gases were improved when compared to a commercial PLA. When compared to the PBS homopolymer, PBS-*ran*-PTDGS copolymers showed a similar performance towards oxygen, with a reduction of up to ~25% in the oxygen transmission rate. Therefore, these materials present a similar or even better barrier to oxygen than the PBS homopolymer. However, the incorporation of thioether linkages resulted in a worsening of the barrier properties of PBS towards carbon dioxide, with transmission rates that doubled those of the PBS homopolymer. This behavior was not due to a decrease in crystallinity, but was explained by the lower chain mobility induced by the higher MW in the copolymers. Notwithstanding, the PBS-*ran*-PTDGS copolymer with 30 mol% thiodiethylene glycol units exhibited the best barrier properties of the whole series, with a better behavior towards oxygen and a similar performance towards carbon dioxide compared to PBS, making it a potential candidate for food packaging applications [88].

Duan et al. synthesized two different sugar-based PBS copolymers, employing two cyclic alditols: isosorbide and 2,3-*o*-isopropylidene-l-threitol. Results showed that isosorbide copolymers presented a ~60% diminishment in oxygen permeability compared to the PBS homopolymer, whereas the threitol-based copolymers reduced the O_2_P by more than 35% when compared to PBS [124].

The opposite behavior was found when PBS was copolymerized with neopentyl glycol and 2-butyl-2-ethyl-propanediol, leading to worse barrier properties than the PBS homopolymer. PBS-*ran*-neopenthyl succinate (PBS-*ran*-PNS) and PBS-*ran*-2-butyl-2-ethyl-propylene succinate (PBS-*ran*-PBEPS) showed a significant increase in gas transmission rates with respect to PBS: more than a 300% increase for CO_2_, ~700% increase in the case of O_2_, and ~800% increase for N_2_ for the PBS-*ran*-PBEPS copolymers; whereas for the PBS-*ran*-PNS copolymer, the gas transmission rates doubled when compared to neat PBS. According to the authors, this behavior was due to a reduction in the crystallinity degree, as gas molecules find it easier to diffuse through the amorphous regions [107]. Despite this, some of these copolymers presented similar or even better barrier properties to those of LDPE, commonly employed in flexible food packages.

### 5.2. PBS-Based Nanocomposites

Besides the variation of mechanical properties, the incorporation of nanofillers within the PBS matrix (and their copolyesters matrices) is a strategy widely employed for the modulation of the barrier properties of this biopolyester. This is a promising approach for enhanced materials for food packaging applications. The reason for this improvement is the physical hindrance to gas molecules due to the presence of fillers in the polymer matrix [125].

The reinforcement of PBS with nanocrystalline cellulose (NCC) and nanoclays enhances the barrier properties of this biopolyester [102]. The study of Xu et al. based on PBS nanocomposites with NCC fillers showed a large improvement in gas barrier properties. With a low amount of NCC (3 wt%), the gas transmission rates were reduced to ~40% and ~60% for water vapor and oxygen, respectively. Furthermore, the addition of 4 wt% of a compatibilizer (methylene diphenyl diisocyanate, MDI) to the PBS with 3 wt% of NCC resulted in a higher reduction of water vapor and oxygen transmission rates to ~63% and ~97%, respectively, compared to the PBS homopolymer [78]. In another example, with PBS-*ran*-PBA nanocomposites prepared by adding unmodified nanoclays (5 wt%), the O_2_P of the PBS-*ran*-PBA was reduced by ~35%. A further reduction of the O_2_P to values of ~50% was obtained with the organic modification of the nanoclays (5 wt%) due to better compatibility with the PBS-*ran*-PBA matrix, which improved the dispersion of the modified clays [114]. Cloisite 30B, an organically modified montmorillonite (OMMT), was studied in PBS/PLA blends (50/50) to improve the PBS barrier properties. A linear relationship between the cloisite content and oxygen/water vapor permeability was found, showing a decrease of ~50% for both gases with 7 wt% of the OMMT [126], in a similar way as for the PLA/PBS-*ran*-PBA and PLA/PBS clay nanocomposites [127], whereas other authors reported a lower reduction of O_2_P in PLA/PBS blends (80/20 w/w) with Cloisite 30B [115]. A similar reduction was achieved for PBS nanocomposites with organically modified layered silicate (OMLS) [112,128], and organomodified beidellite clay nanocomposites [129]. For PBS-*ran*-PBA nanocomposites with native montmorillonite and OMMT, the gas permeabilities drop between 60% and 70% in water-injection-extruded PBS-*ran*-PBA/Cloisite 30B nanocomposites [113].

Similarly, Petchwattana et al. reported that the incorporation of ZnO into PBS films led to a considerable reduction of the gas permeability, as the transmission rates were reduced between ~25% and 30% for water vapor and oxygen, respectively, for the PBS nanocomposite with a 10 wt% of ZnO [79]. The addition of 2 wt% graphene nanoplatelets to PBS involved a ~35% and ~40% reduction of oxygen and water vapor permeabilities, respectively, which was attributed to an increased tortuosity due to the presence of the filler [120]. 

The compatibility between banana starch nanocrystals (SNC) and PBS was evaluated for its application as bionanocomposites for packaging films. Both water vapor and oxygen transmission rates were improved with the incorporation of the modified SNC to PBS, achieving better barrier properties with higher nanocrystals content (9 wt%). These parameters were reduced by ~50% and ~60% for water vapor and oxygen, respectively [130].

Considering PBS/PLA blends (60/40), the study of the influence in barrier properties of two different types of zeolites (5A and 13X) showed that both zeolite nanocomposites achieve lower O_2_P (~60% reduction) and CO_2_P (~40% reduction) when compared to the PBS/PLA blend. This behavior is attributed to the tortuosity and the porosity of zeolites, as O_2_ and CO_2_ molecules are smaller than the zeolite pores. On the other hand, the water vapor permeability increased by ~60% because of the highly polar nature of zeolites [131].

As we have seen within this section, PBS barrier properties can also be modulated by copolymerization with different monomers or by preparing PBS nanocomposites. In PBS copolymers, barrier properties may be enhanced or worsened depending on the nature of the second monomer. The preparation of PBS nanocomposites commonly leads to less permeable materials, as the presence of fillers induces a more tortuous pathway for gas molecules [90,101,102,132,133,134,135]. Although results may differ from one study to another, the general trend has shown that polymeric nanocomposites, and particularly PBS nanocomposites, present better barrier properties than PBS. The aforementioned ways of modulation, linked to the good barrier properties inherent to PBS, compared to other biodegradable polymers (see Figure 7b), make it a potential candidate within this polymer category for certain applications where barrier properties must be taken into consideration, such as food packaging.

## 6. Biodegradation of PBS

Research on biodegradable polymers has become more and more important over the last few years. Nowadays, wastes and landfill accumulation are an increasing concern worldwide, and biodegradable polymers emerge as a promising alternative for its reduction [136,137]. The situation is especially dramatic regarding plastic waste into the oceans, where plastic fragments within the range of a few micrometers to several centimeters end up in the ocean and cause significant damage to the local wildlife and the ecosystems [7,138,139]. 

PBS, as already mentioned in this review, is a biodegradable polyester that can effectively decompose into water and carbon dioxide (CO_2_). PBS can be included in the fossil-based biodegradable polymers, although many advances have been made in the field of bio-based PBS (Section 2). Because of this, PBS and many of its copolymers can be biodegraded (i.e., naturally occurring enzymes and microorganisms) despite its monomers (SA and BD) being mainly produced from petroleum derivatives. Several reviews can be found in the literature that discuss the most common biodegradation routes for biodegradable polymers, such as PCL, PLA, or PHAs [34,140]. Nevertheless, less information regarding PBS biodegradation is available, as research is still ongoing. PBS degradation methods include hydrolytic degradation, enzymatic degradation, and biodegradation in environmental conditions, such as burial, activated sludge, and compost [32].

### 6.1. Hydrolytic Degradation

One of the most common mechanisms of polymer degradation is hydrolytic degradation. In this case (and in the case of enzymatic degradation), the degradation rate depends on PBS crystallinity. Hydrolytic degradation occurs faster in the lower density amorphous regions, facilitating water penetration. This phenomenon causes an increase in the overall degree of crystallinity due to the faster degradation of amorphous domains (that can crystallize once degraded) compared to the more crystalline ones [141]. Some authors report no variation in weight for PBS when exposed to hydrolytic degradation [142], while others report low weight loss [143,144]. One study reported a ~31% weight loss for PBS after 24 weeks of hydrolytic degradation at 37 °C. This result could be explained due to the relatively low crystallinity (~56%, as determined by DSC) of the PBS used [145]. The pH of the media is also an important parameter that must be taken into account. Morales-Huerta et al. reported a 10% weight loss for hydrolytic degradation at pH = 7.4 after 30 days, whereas the weight loss increased to values higher than 25% for a pH = 2.0 media [146].

As can be deduced from different studies, PBS can be effectively degraded by the hydrolysis of the ester bonds, achieving different results depending on many different parameters involved, such as the synthesis method, MW, crystallinity, or the experimental conditions of the biodegradation assays.

### 6.2. Enzymatic Degradation

So far, enzymatic degradation is regarded as one of the most attractive and effective methods for the biodegradation of biopolyesters. The main reason is the presence of labile ester bonds in the chemical structures of biopolyesters, where enzymes can attack [99]. Then, the enzymatic degradation process usually starts with the attachment of the enzyme on the surface, and hydrolysis proceeds via surface erosion. Among all the different types and families of enzymes that can effectively biodegrade PBS and its copolymers, some examples are included in this manuscript. Table 1 shows various enzymes and different experimental conditions for PBS enzymatic biodegradation. 

Enzymatic degradation assays for biopolyesters and PBS are commonly carried out at physiological temperature (i.e., 37 °C) [146,149,152]. However, it has been demonstrated that this degradation method is favored at a temperature close to *T_m_* (the PBS melting temperature is above 100 °C) [157]. Some authors have reported low weight loss values for PBS homopolymer at different experimental conditions, reaching a 3.5% weight loss after 12 days in the presence of *Pseudomonas cepacia* lipase [157], or even lower [151,153]. The low degree of degradation obtained could be attributed to the high crystallinity of this polymer compared to other aliphatic polyesters [157]. Other studies carried out under different experimental conditions report much higher degradation rates. For example, for enzymatic degradation assays employing cutinases, weight losses reach almost 100% in just 12 h [149], as seen in Table 1. An interesting study developed by Shi et al. showed the influence of two different enzymes (*Fusarium solani* cutinase and *Candida antarctica* lipase B, CALB) in the degradation rate of PBS. They found that the PBS degradation rate was much faster by the action of cutinase. PBS degraded in the presence of cutinase reached ~50% weight loss in 4 h, whereas those degraded in the presence of lipase reached ~20% weight loss over the same time. For both cases, a nearly total decomposition was achieved after 26 h [150].

**Figure 8:**
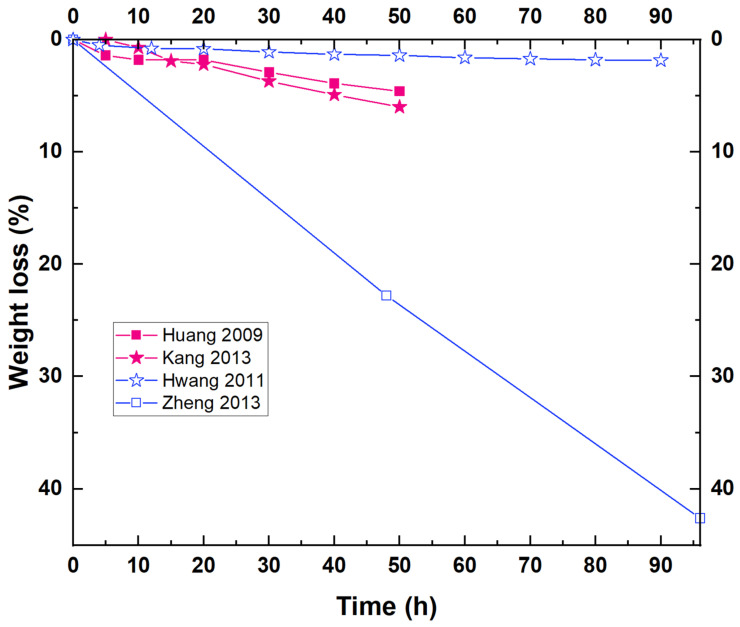
Weight loss curves corresponding to various enzymatic degradation studies of PBS by the action of a lipase from *Pseudomonas cepacia*. Experimental conditions differ from one study to another, although the same enzyme is used.

Figure 8 shows the results corresponding to different biodegradation studies where the weight loss (%) of PBS in the presence of lipase from *Pseudomonas cepacia* has been reported. As can be observed in Figure 8, only one study shows a relatively high weight loss of PBS with this enzyme (higher than 40% in 100 h) [71]. For the rest, the weight loss reached after several hours in contact with *Pseudomonas cepacia* is very low (less than 6%), which could be attributed, in part, to the low concentration of the enzyme employed for some of the studies [152,154,156].

### 6.3. PBS-Based Copolymers: Hydrolytic and Enzymatic Degradation

In the case of PBS copolymers, different (and opposite) results are reported depending on the nature of the second comonomer. For instance, the biodegradability of aromatic polyesters is less favored than in the case of aliphatic polyesters such as PBS [146]. Thus, incorporating a second comonomer in the structure of PBS could favor or prevent the degradation of the polyester, attending to the nature of the second constituent. 

Hydrolytic and enzymatic degradation of PBS-*ran*-PBFur copolyesters have been determined by placing 60:40 and 40:60 copolymers in a pH = 2.0 or pH = 7.4 medium at 37 °C [146]. Firstly, the enzymatic degradation was more effective than hydrolysis, reaching 15–20% of weight loss versus 3–5% for the latter (hydrolysis) after 30 days. Furthermore, the behavior of the copolyesters was more similar to that of PBS in the case of enzymatic degradation. Regarding the acidic medium, the results were in between enzymatic and hydrolytic degradation, but far away from those obtained for the PBS homopolymer, as the homopolymer achieved a 30% of weight loss, compared to the 10–15% weight loss of the copolymers. 

Han et al. studied the enzymatic degradation behavior for different poly(butylene succinate-*ran*-butylene 2-methylsuccinate) (PBS-*ran*-PBMS) copolyesters, reporting higher degradation rates for those copolymers with a higher PBMS content. Considering the copolymer with 20% mol in PBMS, the hydrolytic degradation (without the enzyme) showed a negligible weight loss compared to that of the enzymatic degradation (amano lipase from *Pseudomonas fluorescens*), achieving a 30% weight loss in 300 h [155].

The copolymerization of PBS with salicylic acid was studied as an attempt to produce polymer films with potential applications in agricultural applications. Enzymatic degradation assays carried out in the presence of *Candida antarctica* lipase B (CALB) showed very low degradation after 20 days (~1.5 wt% for neat PBS); however, the addition of salicylic acid increased this value up to ~3.5 wt% [158]. 

In the case of the enzymatic hydrolysis of PBS and PBS-*ran*-PBA copolymers in the presence of *Candida cylindracea* lipase [159], the highest degradation was obtained for the copolyesters containing 25% and 50% mol of butylene succinate, reaching 20% and 30% of weight loss, respectively, after 90 h. It is necessary to remark that the enzymatic degradation is not affected by the MW; hence, similar results are obtained for low MW (i.e., *M_w_* of 6300 g/mol) and high MW (i.e., *M_w_* of 29,000 g/mol) [160]. 

### 6.4. Biodegradation in Environmental Conditions

Although enzymatic hydrolysis (laboratory conditions) has shown satisfactory results for PBS biodegradation, this biopolyester commonly degrades in environmental conditions [161]. The study of the biodegradation of PBS under environmental conditions will give an idea for the implementation of PBS in agricultural applications such as mulching films [53,100,162]. PE films are commonly employed for this application, being an effective method for promoting plant growth during the cold seasons (i.e., spring and autumn). The problem here is the recyclability of the PE film due to the contamination caused to the soil itself, so a biodegradable film is required, and PBS is a suitable candidate to solve this issue [163]. 

The experiments for this type of biodegradation are usually carried out following different standards from international organizations (ISO, ASTM, and EU). Because of this, the definition of more experimental parameters is required as compared to enzymatic and hydrolytic assays. As the conditions and parameters differ from one study to another (as well as the soil employed for the tests and the microorganisms content in the soil), biodegradation in environmental conditions covers a wide range of variable results [164]. Below we summarize the representative results corresponding to biodegradation studies carried out under environmental conditions for PBS homopolymer and copolymers, with special focus on PBS composites with biofillers (PBS-based biocomposites).

#### 6.4.1. PBS Homopolymer and PBS-Based Copolymers

PBS biodegradation in environmental conditions usually takes more time as compared to enzymatic/hydrolytic PBS degradation. Kim et al. reported a low degradation of PBS when exposed to environmental degradation (below 8% weight loss after 120 days) [165]. Similar trends have been obtained by Huang et al. (below 3% weight loss in 100 days) [166] and other reports [137]. However, the study of PBS biodegradation in a controlled compost at 58 °C (based on ISO 14855-2) showed that PBS powder biodegradation reached 60% weight loss in 40 days and increased to 80% in less than 80 days. These results are highly promising, opening a path for the establishment of experimental protocols to determine the environmental biodegradation of this aliphatic polyester [136]. Kunioka et al. also reported very high biodegradation rates for PBS in powder form, reaching almost 80% weight loss in less than 80 days [136]. These outstanding results are explained as the PBS was tested in powder form, which differs from the tensile specimens commonly used to determine environmental biodegradation.

The biodegradation of PBS and PBS-*ran*-PBA copolymers subjected to different environments, as biodegradation in compost, soil, and artificial weathering, has been reported. For the artificial weathering, both polymers were submitted to UVA radiation and artificial rain, whereas in soil and compost experiments, no radiation was employed. For the first assay, a ~30% weight loss was achieved for PBS in 24 weeks (~50% in the case of the PBS-*ran*-PBA copolymer). In contrast, biodegradation in soil and artificial weathering showed negligible degradation for PBS, while PBS-*ran*-PBA presented a ~20% weight loss for the biodegradation in soil experiment and negligible for artificial weathering [98]. In another study, the biodegradation of PBS-*ran*-PBFur copolymers in compost at 58 °C showed the best results for the 20 mol% of furanoate composition, achieving an almost 100% of degradation in 80 days [87]. 

#### 6.4.2. PBS-Based Biocomposites

The presence of fillers in PBS biocomposites has been widely studied to modulate the degradation in environmental conditions, as in the case of other types of degradation and thermomechanical and barrier properties, as we have seen in previous sections. Special cases are natural fillers, that, in addition to being easily biodegraded, can potentially increase the degradation rate of PBS. Table 2 includes several examples of biodegradation studies carried out under environmental conditions for different PBS-based biocomposites. Among all the examples presented in this table, some interesting results will be commented on below. As a general idea to consider, the trend shows that PBS-based biocomposites degrade faster than neat PBS.

**Figure 9:**
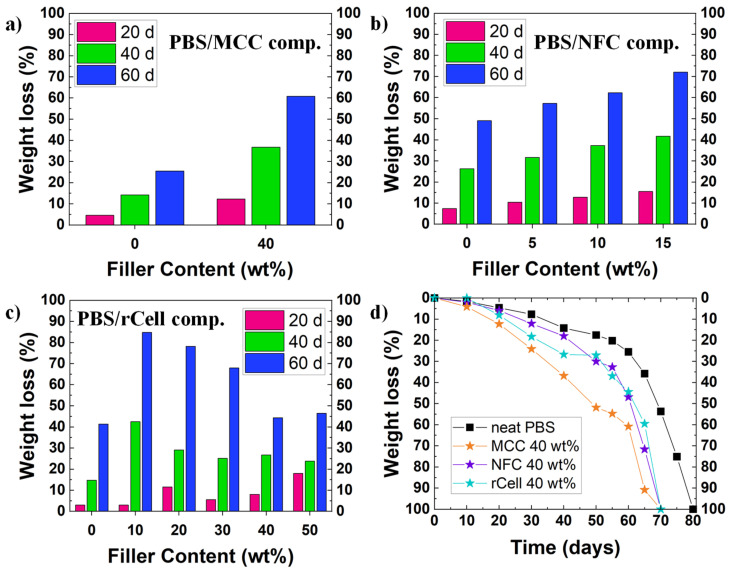
Biodegradation in environmental conditions for several PBS/cellulose-based biocomposites: influence of filler content in weight loss for (**a**) PBS/MCC composites, (**b**) PBS/NFC composites, and (**c**) PBS/rCell composites; (**d**) variation of biodegradation rates for PBS and the three PBS/cellulose-based biocomposites. In Figure 9a–c, weight losses at 20, 40, and 60 days are represented.

Although the experimental conditions differ from one study to another, it has been observed that cellulose fillers (micro- or nano-sized) achieve one of the best results for PBS degradation. Platnieks et al. have studied many different PBS/cellulose-based composite films: microcrystalline cellulose (MCC) [167], nanofibrillated cellulose (NFC) [153,160], and recycled cellulose from TetraPak^®^ [175]. The experimental conditions were similar for all the studies, employing a simulated compost under aerobic conditions at 58 °C, with a slightly acidic medium (pH = 5.7–6.5) and a water content of 50% or higher. The authors found that, although almost every sample was completely disintegrated within 75–80 days, PBS-biocomposites degraded 5–10 days earlier than neat PBS films. In general, the degradation rate was faster at higher filler content. However, for the PBS/rCell biocomposite with the highest content in rCell, the degradation rate was faster in the early stages of the assay, whereas it slowed down during the course of the experiment (see Figure 9a–c). If we compare the results corresponding to the PBS biocomposites with high filler content (i.e., 40 wt%), it was found that the PBS/MCC composite degraded faster than the other two biocomposites (i.e., PBS/NFC and PBS/rCell) which present a similar behavior, being much faster than the biodegradation of neat PBS (see Figure 9d).

Other PBS biocomposites include rubberwood powder (RWP) from sawdust wastes as a natural filler (lignocellulosic nature). The environmental degradation of these PBS/RWP biocomposites was studied, showing a ~10 wt% weight loss after 60 days of soil burial testing. This behavior was attributed to the decrease in the crystallinity of the PBS biocomposites with the increasing content of RWP [137]. In another example, the biodegradation of the PBS biocomposites with rice husk flour (RHF) and wood flour (WF) in soil burial testing showed a 10 wt% degradation for the RHF composites after 120 days [165]. In both cases, the weight loss was directly related to the biocomposite content, increasing with the filler content. 

In this section, we have discussed the biodegradation of PBS and PBS-based materials in environmental conditions, which could lead to the employment of this biopolyester as mulching films for agricultural purposes. As has been commented on within this section, PBS presents a slower degradation rate when subjected to environmental conditions than enzymatic and hydrolysis conditions. However, its effective disintegration in the environment opens the door to many interesting applications where the material should not remain in the environment but needs to be usable for a certain period of time, as for the aforementioned agricultural uses such as plastic mulching films [99]. 

## 7. Conclusions and Future Perspectives

This paper focused on reviewing the current strategies to modulate the thermo-mechanical, barrier, and biodegradation properties of PBS. The modulation of the high crystallization and low degradation rates are desired for the commercial applications of PBS. With this aim, we revised the random copolymerization of PBS. PBS random copolyesters displayed a rich crystallization behavior since they can crystallize in three crystallization modes: isomorphism, comonomer exclusion, and isodimorphism. Independently of the crystallization mode, the PBS-rich compositions can crystallize, in most cases with a depression of the transition temperatures and the crystallinity. These thermal properties depression, on the one hand, extends the applications of PBS copolymers to a broad range of final use temperatures, and, on the other hand, represents the desired reduction of the high crystallinity of PBS, which influences its mechanical properties and degradation, among others. As a result, PBS-based copolymers are promising materials, as copolymerization is an excellent route to tune the properties of PBS. 

The modulation of the mechanical and barrier properties can also be achieved by using fillers (micro- and nanosized particles) as additives, creating composite materials based on PBS. Currently, in this revision, we have found that several fillers are being investigated to tailor the PBS properties, including inorganic and organic fillers with bio-based nanofillers. One of the most investigated bio-based nanofillers is cellulose nanowhiskers due to the possibility of achieving fully biodegradable composite materials. The mechanical and barrier properties improvements are generally attained at a low filler content. Nevertheless, the design of composite PBS materials and the study of the resulting properties must be done on a one-by-one case because the final performance depends on many variables (filler dimensionality, interaction between the polymer matrix and the filler, and many others). 

This review highlights the wide variety of existing methodologies in the literature to measure PBS degradation through different routes (hydrolytic, enzymatic, and soil). Enzymatic degradation constitutes one of the most promising routes to biodegrade this polymer. However, the results reported in the literature are highly influenced by the type and concentration of the enzyme employed in the experiments and the experimental conditions (i.e., temperature), making it difficult to establish common trends in the enzymatic degradation of PBS. It is then foreseeable and necessary to develop standard protocols or set up general experimental methodologies to measure enzymatic PBS degradation to support the rapid development of PBS and its copolymers.

Future research is needed to continue tailoring PBS properties. From a synthesis point of view, implementing sustainable and efficient polymerization routes from bio-based monomers will be required. In this line, “green catalysts” (i.e., enzymes, revised in this contribution) emerge as a strong alternative, intending to achieve high-MW PBS with comparable properties to those obtained from traditional polycondensation routes. For the modulation of PBS properties, investigating the structure–properties relationship of PBS and PBS-based materials and its relation to processing is necessary in aiming to design commercial applications for this biopolyester. The modulation strategies should strive to achieve the desired changes, e.g., decreasing the crystallinity, without affecting the biodegradable character of the PBS. 

## Figures and Tables

**Table 1:** Classification of enzymes by families for different PBS enzymatic degradation studies.

Family	Enzyme	Substrate	Experimental Conditions	Results	Reference
Cutinase	*Fusarium solani*	PBS films 30 × 10 × 0.1 mm^3^	pH = 8.0 at 40 °C, 20 µg/mL	100% weight loss in 6 h	[147]
Cutinase	*Pichia pastoris*	PBS films 30 × 10 × 0.5 mm^3^	pH = 7.4 at 37 °C, 0.15 mg/mL	100% weight loss in 12 h	[148]
Cutinase	*Fusarium solani*	PBS films 30 × 10 × 0.1 mm^3^	pH = 7.4 at 37 °C, 10 mg/mL	98.4% weight loss in 12 h	[149]
Cutinase	*Fusarium solani*	PBS films 30 × 10 × 0.5 mm^3^	pH = 7.2 at 37 °C, 18 U/mL	~100% weight loss in 26 h	[150]
Lipase	*Candida antarctica* (CALB)	PBS films 30 × 10 × 0.5 mm^3^	pH = 7.2 at 45 °C, 18 U/mL	95.1% weight loss in 26 h	[150]
Lipase	*Candida rugosa*	PBS films 10 × 10 × 0.5 mm^3^	pH = 7.4 at 30 °C, 0.1 mg/mL	2% weight loss after 7 weeks	[151]
Lipase	*Pseudomonas cepacia*	PBS films 20 × 30 × 0.3 mm^3^	pH = 8.0 at 40 °C, 0.06 mg/mL	2% weight loss after 90 h	[152]
Lipase	*Candida antarctica* (CALB) N435	PBS films 30 × 10 mm^2^	pH = 7.4 at 37 °C, 1.2 mg/mL	1.8% weight loss after 90 h	[153]
Lipase	Porcine pancreas	PBS films 30 × 10 mm^2^	pH = 7.4 at 37 °C, 0.8 mg/mL	0.9% weight loss after 90 h	[153]
Lipase	*Pseudomonas cepacia*	PBS films 10 × 10 × 0.1 mm^3^	pH = 6.86 at 45 °C, 0.22 mg/mL	4.6% weight loss after 50 h	[154]
Lipase	Porcine pancreas	PBS film discs 10 × 10 × 0.2 mm^3^	pH = 7.4 at 37 °C, 1 mg/mL	21% weight loss after 30 days	[146]
Lipase	*Pseudomonas fluorescens*	PBS films 10 × 10 × 0.2 mm^3^	pH = 7.3 at 37 °C, 2 mg/mL	No visible degradation after 300 h	[155]
Lipase	*Pseudomonas cepacia*	PBS films 10 × 10 × 0.1 mm^3^	pH = 6.86 at 45 °C, 0.53 mg/mL	100% weight loss after 288 h	[71]
Lipase	*Pseudomonas cepacia*	PBS film discs 20 × 20 × 0.05 mm^3^	pH = 7.4 at 37 °C, 1 mg/mL	6% weight loss after 50 h	[156]
Lipase	*Rhizopus delemar* and *Pseudomonas cepacia*	PBS film discs 50 × 50 × 2 mm^3^	pH = 7.2 at 30 °C, 0.09 & 0.01 mg/mL	2% weight loss after 360 h	[66]

**Table 2:** Different biodegradation studies of PBS and PBS biocomposites carried out in environmental conditions.

Filler	Filler Content	Experimental Conditions	Results (Weight Loss)	Reference
Rubberwood powders (RWP)	0–40 wt%	60 days, no UV radiation, water control each 48 h	<1% (PBS) 2–10% (PBS/RWP)	[137]
Rice husk flour (RHF) and wood flour (WF)	0–40 wt%	4 months	7% (PBS) 8–12% (PBS/RHF and PBS/WF)	[165]
Sugarcane rind fiber (SRF)	0–15 wt%	100 days, natural soil in cropland, water control each 24 h	2.5% (PBS) 10–20% (PBS/SRF)	[166]
Microcrystalline cellulose (MCC) and nanofibrillated cellulose (NFC)	0–40 wt%	75 days, simulated compost, 58 °C, pH = 5.7–6.3, 50 wt% water content	100% in 75–80 days (PBS) 100% in 65–70 days (PBS/MCC and PBS/NFC)	[167]
Cotton fiber (CF)	0–40 wt%	Based on ISO 14855-2 100 days, 58 °C, 10 mL/min air flow	~60% (PBS) ~90% (PBS/CF)	[168]
Rice husk flour (RHF)	0–40 wt%	Based on ASTM D 6003-96 80 days, 30 °C, pH = 7, 50–60 wt% water content	~12% (PBS) 13–18% (PBS/RHF)	[169]
Jute fiber (JF)	0–30 wt%	180 days, compost soil, 30 °C, constant water control	31.4% (PBS) 47.3–62.5% (PBS/JF)	[170]
Abaca fiber (AF)	10 wt%	180 days, black soil and leaf mold for gardening, 25–30 °C, water control each 48 h	~30% (PBS) ~50% (PBS/AF)	[171]
Soy, canola, and corn gluten meals (SM, CM, CGM) and switchgrass (SG)	25 wt%	Based on ASTM D6400 200 days, 3 month-old compost, 58 °C, pH = 7–8, 50–55 wt% water content	~95% (PBS) ~85% (PBS/SG) 90–95% (PBS/SM, PBS/CM and PBS/CGM)	[172]
Organically modified montmorillonite (OMMT)	0–10 wt%	180 days, natural compost, 30 °C, pH = 7.46, 60–70 wt% water content	~9% (PBS) ~3.5–5% (PBS/OMMT)	[173]
Nanofibrillated cellulose (NFC) and recycled cellulose (rCell)	0–15 wt% (PBS/NFC) 0–50 wt% (PBS/rCell)	80 days, 58 °C, pH = 5.7–6.5, >50 wt% water content	~80% (PBS) ~85–92% (PBS/NFC) 100% in 70 days (PBS/rCell)	[174,175]
Pistachio shell flour (PSF)	0–30 wt%	Based on ISO 20200 112 days, compost, 58 °C, 55 % relative humidity	~18% (PBS) ~14–17.5% (PBS/PSF)	[176]

## Abbreviations

**Symbol/Acronym in the Text**	**Definition**
$\Delta H_{m}$	Melting enthalpy
$\Delta H_{m}^{0}$	Equilibrium melting enthalpy
*E*	Young’s modulus
*ε*	Elongation at break
∅	Weight fraction
*M_n_*	Number average molecular weight
*M_w_*	Weight average molecular weight
*T_c_*	Crystallization temperature
*T_m_*	Melting temperature
*X_c_*	Degree of crystallinity
AF	Abaca fiber
ASTM	American Society for Testing and Materials
BD	1,4-butanediol
BS	Butylene succinate
C25A	Cloisite^®^ 25A
CALB	*Candida antarctica* lipase B
CF	Cotton fiber
CGM	Corn gluten meal
CL	Caprolactone
CM	Canola meal
CNC	Cellulose nanocrystals
CO_2_P	Carbon dioxide permeability
DES	Diethyl succinate
DMAz	Dimethylazelate
DMS	Dimethyl succinate
DSC	Differential scanning calorimetry
eROP	Enzymatic ring-opening polymerization
EVOH	Ethylene vinyl alcohol
FHT	Fluorohectorite
HA	Hydroxyapatite
HDPE	High-density polyethylene
HMDI	Hexamethylene diisocyanate
INF	Isora nanofibers
ISO	International Standardization Organization
JF	Jute fiber
KFP	Konjac fly powders
LCP	Liquid crystal polymer
LDPE	Low-density polyethylene
MCC	Microcrystalline cellulose
MDI	Methylene diphenyl diisocyanate
MMT	Montmorillonite
MW	Molecular weight
NCC	Nanocrystalline cellulose
NFC	Nanofibrillated cellulose
NR	Natural rubber
O_2_P	Oxygen permeability
OMLS	Organically modified layered silicate
OMMT	Organically modified montmorillonite
OPMF	Oil palm mesocarp fibers
PAN	Polyacrylonitrile
PBF	Poly(butylene fumarate)
PBMS	Poly(butylene 2-methylsuccinate)
PBS	Poly(butylene succinate)
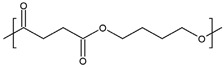
PBSu	Poly(butylene succinate)
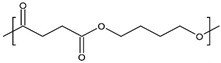
PBT	Polybutylene terephthalate
PcBS	Poly(*cis*-butene succinate)
PCL	Poly(ε-caprolactone)
PDMS	Polydimethylsiloxane
PE	Polyethylene
PEG	Poly (ethylene glycol)
PEMA	Poly (ethyl methacrylate)
PET	Poly (ethylene terephthalate)
PGA	Poly(glycolide)
PHAs	Polyhydroxyalkanoates
PHB	Polyhydroxybutyrate
PHBV	Poly(3-hydroxybutyrate-*co*-3-hydroxyvalerate)
PHS	Poly(hexamethylene succinate)
PLA	Poly(lactic acid)
PMP	Polymethylpentene
PP	Polypropylene
PPO	Polyphenylene oxide
PPS	Poly(propylene succinate)
PRHB	Poly((r)-3-hydroxybutyrate)
PS	Polystyrene
PSF	Pistachio shell flour
PTFE	Polytetrafluoroethylene
PTMS	Poly(tetramethylene succinate)
PTMSP	Poly(1-trimethylsilyl-1-propyne)
PVA	Polyvinyl alcohol
PVC	Polyvinyl chloride
PVDC	Polyvinylidene chloride
PVDF	Polyvinylidene fluoride
rCell	Recycled cellulose
RHF	Rice husk flour
RT	Room temperature
RWP	Rubberwood powders
SA	Succinic acid
SAP	Saponite
SG	Switchgrass
SM	Soy meal
SNC	Starch nanocrystals
SRF	Sugarcane rind fiber
TNBT	Titanium (IV) butoxide
TTIP	Titanium (IV) isopropoxide
ULDPE	Ultra-low-density polyethylene
UVA	Ultraviolet A
WAXS	Wide Angle X-ray Scattering
WF	Wood flour
**Copolymers cited within the review.**	
**Acronym in the Text**	**Definition and Chemical Structure**
PBS-*co*-PBF	Poly(butylene succinate-*co*-butylene fumarate)
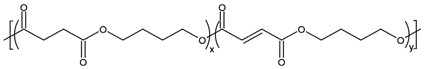
PBS-*ran*-PBA	Poly(butylene succinate-*ran*-butylene adipate)
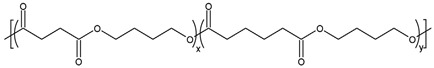
PBS-*ran*-PBAz	Poly(butylene succinate-*ran*-butylene azelate)
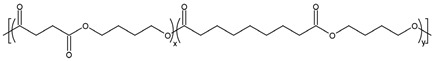
PBS-*ran*-PBEPS	Poly(butylene succinate-*ran*-2-butyl-2-ethyl-propylene succinate)
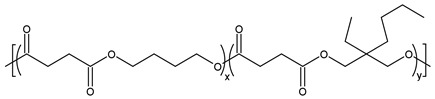
PBS-*ran*-PBF	Poly(butylene succinate-*ran*-butylene fumarate)
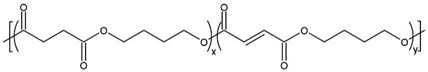
PBS-*ran*-PBF-*ran*-PBA	Poly(butylene succinate-*ran*-butylene fumarate-*ran*-butylene adipate)
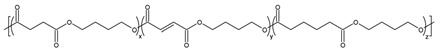
PBS-*ran*-PBFur	Poly(butylene succinate-*ran*-butylene furanoate)
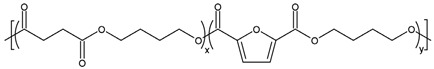
PBS-*ran*-PBMS	Poly(butylene succinate-*ran*-butylene 2-methylsuccinate)
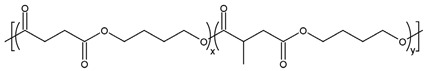
PBS-*ran*-PBT	Poly(butylene succinate-*ran*-butylene terephthalate)
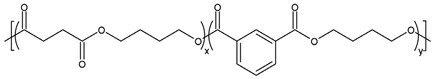
PBS-*ran*-PcBS	Poly(butylene succinate-*ran*-*cis*-butene succinate)
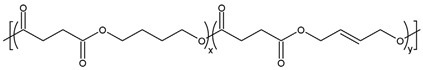
PBS-*ran*-PCL	Poly(butylene succinate-*ran*-ε-caprolactone)
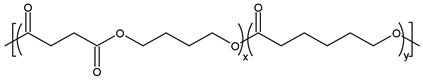
PBS-*ran*-PDMSu	Poly(butylene succinate-*ran*-decamethylene succinate)
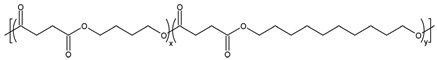
PBS-*ran*-PHS	Poly(butylene succinate-*ran*-hexamethylene succinate)
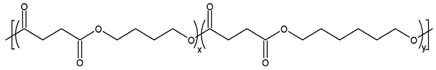
PBS-*ran*-PIS	Poly(butylene succinate-*ran*-isosorbide succinate)
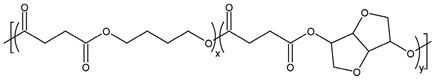
PBS-*ran*-PNS	Poly(butylene succinate-*ran*-neopenthyl succinate)
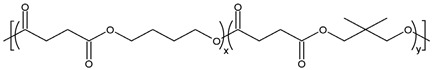
PBS-*ran*-PPS	Poly(butylene succinate-*ran*-propylene succinate)
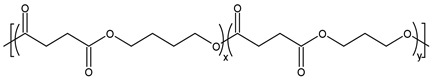
PBS-*ran*-PTDGS	Poly(butylene succinate-*ran*-thiodiethylene glycol succinate)

## References

[1] Plastics Europe (2019). Plastics—The Facts 2019: An Analysis of European Plastics Production, Demand and Waste data.

[2] Plastics Europe (2021). Plastics—The Facts 2021: An Analysis of European Plastics Production, Demand and Waste Data.

[3] Geyer R., Jambeck J.R., Law K.L. (2017). Production, use, and fate of all plastics ever made. Sci. Adv..

[4] Rosenboom J.-G., Langer R., Traverso G. (2022). Bioplastics for a circular economy. Nat. Rev. Mater..

[5] Rafiqah S.A., Khalina A., Harmaen A.S., Tawakkal I.A., Zaman K., Asim M., Nurrazi M.N., Lee C.H. (2021). A review on properties and application of bio-based poly(butylene succinate). Polymers.

[6] Chinthapalli R., Skoczinski P., Carus M., Baltus W., De Guzman D., Käb H., Raschka A., Ravenstijn J. (2019). Biobased Building Blocks and Polymers—Global Capacities, Production and Trends, 2018–2023. Ind. Biotechnol..

[7] Chen G.Q., Patel M.K. (2012). Plastics derived from biological sources: Present and future: A technical and environmental review. Chem. Rev..

[8] Jambeck J.R., Ji Q., Zhang Y.-G., Liu D., Grossnickle D.M., Luo Z.-X. (2015). Plastic waste inputs from land into the ocean. Science.

[9] ten Brink P., Schweitzer J.-P., Watkins E., Janssens C., De Smet M., Leslie H., Galgani F. (2018). Circular economy measures to keep plastics and their value in the economy, avoid waste and reduce marine litter. Econ. Discuss. Pap..

[10] Abhilash M., Thomas D. (2017). Biopolymers for Biocomposites and Chemical Sensor Applications. Biopolymer Composites in Electronics.

[11] Zini E., Scandola M. (2011). Green Composites: An Overview. Polym. Compos..

[12] Saratale R.G., Cho S.K., Saratale G.D., Kadam A.A., Ghodake G.S., Kumar M., Bharagava R.N., Kumar G., Kim D.S., Mulla S.I. (2021). A comprehensive overview and recent advances on polyhydroxyalkanoates (PHA) production using various organic waste streams. Bioresour. Technol..

[13] European Bioplastics (2019). Bioplastics Market Data 2019. Global Production Capacities of Bioplastics 2019–2024.

[14] Ratshoshi B.K., Farzad S., Görgens J.F. (2021). Techno-economic assessment of polylactic acid and polybutylene succinate production in an integrated sugarcane biorefinery. Biofuels Bioprod. Biorefining.

[15] Ioannidou S.M., Ladakis D., Moutousidi E., Dheskali E., Kookos I.K., Câmara-Salim I., Moreira M.T., Koutinas A. (2022). Techno-economic risk assessment, life cycle analysis and life cycle costing for poly(butylene succinate) and poly(lactic acid) production using renewable resources. Sci. Total Environ..

[16] Ni Y., Richter G.M., Mwabonje O.N., Qi A., Patel M.K., Woods J. (2021). Novel integrated agricultural land management approach provides sustainable biomass feedstocks for bioplastics and supports the UK’s “net-zero” target. Environ. Res. Lett..

[17] Gigli M., Fabbri M., Lotti N., Gamberini R., Rimini B., Munari A. (2016). Poly(butylene succinate)-based polyesters for biomedical applications: A review in memory of our beloved colleague and friend Dr. Lara Finelli. Eur. Polym. J..

[18] Sander M. (2019). Biodegradation of Polymeric Mulch Films in Agricultural Soils: Concepts, Knowledge Gaps, and Future Research Directions. Environ. Sci. Technol..

[19] Wu F., Misra M., Mohanty A.K. (2021). Challenges and new opportunities on barrier performance of biodegradable polymers for sustainable packaging. Prog. Polym. Sci..

[20] Platnieks O., Gaidukovs S., Kumar Thakur V., Barkane A., Beluns S. (2021). Bio-Based Poly (Butylene Succinate): Recent Progress, Challenges and Future Opportunities. Eur. Polym. J..

[21] Larrañaga A., Lizundia E. (2019). A review on the thermomechanical properties and biodegradation behaviour of polyesters. Eur. Polym. J..

[22] Su S., Kopitzky R., Tolga S., Kabasci S. (2019). Polylactide (PLA) and Its Blends with Poly(butylene succinate) (PBS): A Brief Review. Polymers.

[23] Sionkowska A. (2011). Current research on the blends of natural and synthetic polymers as new biomaterials: Review. Prog. Polym. Sci..

[24] Soccio M., Dominici F., Quattrosoldi S., Luzi F., Munari A., Torre L., Lotti N., Puglia D. (2020). PBS-based green copolymer as efficient compatibilizer in Thermoplastic inedible Wheat Flour/Poly (Butylene Succinate) Blends. Biomacromolecules.

[25] Zhang Q., Song M., Xu Y., Wang W., Wang Z., Zhang L. (2021). Bio-based polyesters: Recent progress and future prospects. Prog. Polym. Sci..

[26] Safari M., Otaegi I., Aramburu N., Guerrica-Echevarria G., Martínez de Ilarduya A., Sardon H., Müller A.J. (2021). Synthesis, structure, crystallization and mechanical properties of isodimorphic PBS-ran-PCL copolyesters. Polymers.

[27] Sandoval A.J., Fernández M.M., Candal M.V., Safari M., Santamaria A., Müller A.J. (2022). Rheology and Tack Properties of Biodegradable Isodimorphic Poly(butylene succinate)-Ran-Poly(ε-caprolactone) Random Copolyesters and Their Potential Use as Adhesives. Polymers.

[28] Burk M.J., Van Dien S.J., Burgard A., Niu W. (2011). Compositions and Methods for the Biosynthesis of 1,4-Butanediol and Its Precursors. U.S. Patent.

[29] Díaz A., Katsarava R., Puiggalí J. (2014). Synthesis, properties and applications of biodegradable polymers derived from diols and dicarboxylic Acids: From Polyesters to poly(ester amide)s. Int. J. Mol. Sci..

[30] Tserki V., Matzinos P., Pavlidou E., Vachliotis D., Panayiotou C. (2006). Biodegradable aliphatic polyesters. Part I. Properties and biodegradation of poly(butylene succinate-co-butylene adipate). Polym. Degrad. Stab..

[31] Tserki V., Matzinos P., Pavlidou E., Panayiotou C. (2006). Biodegradable aliphatic polyesters. Part II. Synthesis and characterization of chain extended poly(butylene succinate-co-butylene adipate). Polym. Degrad. Stab..

[32] Xu J., Guo B.-H. (2010). Microbial succinic acid, its polymer poly(butylene succinate), and applications. Plastics from Bacteria: Natural Functions and Applications.

[33] Xu J., Guo B.H. (2010). Poly(butylene succinate) and its copolymers: Research, development and industrialization. Biotechnol. J..

[34] Luyt A.S., Malik S.S. (2019). Can biodegradable plastics solve plastic solid waste accumulation?. Plastics to Energy: Fuel, Chemicals, and Sustainability Implications.

[35] Zhu Q.Y., He Y.S., Zeng J.B., Huang Q., Wang Y.Z. (2011). Synthesis and characterization of a novel multiblock copolyester containing poly(ethylene succinate) and poly(butylene succinate). Mater. Chem. Phys..

[36] Zhou H., Wang X., Du Z., Li H., Yu K. (2015). Preparation and characterization of chain extended Poly(butylene succinate) foams. Polym. Eng. Sci..

[37] Fujimaki T. (1998). Processability and properties of aliphatic polyesters, “BIONOLLE”, synthesized by polycondensation reaction. Polym. Degrad. Stab..

[38] Huang C.Q., Luo S.Y., Xu S.Y., Zhao J.B., Jiang S.L., Yang W.T. (2010). Catalyzed chain extension of poly(butylene adipate) and poly(butylene succinate) with 2,2′-(1,4-phenylene)-bis(2-oxazoline). J. Appl. Polym. Sci..

[39] Zhao J.B., Li K.Y., Yang W.T. (2007). Chain extension of polybutylene adipate and polybutylene succinate with adipoyl- and terephthaloyl-biscaprolactamate. J. Appl. Polym. Sci..

[40] Zhao J.B., Wu X.F., Yang W.T. (2004). Synthesis of aliphatic polyesters by a chain-extending reaction with octamethylcyclotetrasilazane and hexaphenylcyclotrisilazane as chain extenders. J. Appl. Polym. Sci..

[41] Ishii M., Okazaki M., Shibasaki Y., Ueda M., Teranishi T. (2001). Convenient synthesis of aliphatic polyesters by distannoxane-catalyzed polycondensation. Biomacromolecules.

[42] Zhu C., Zhang Z., Liu Q., Wang Z., Jin J. (2003). Synthesis and Biodegradation of Aliphatic Polyesters from Dicarboxylic Acids and Diols. J. Appl. Polym. Sci..

[43] Jie S. (2007). Synthesis and Characterization of High Relative Molecular Mass Poly(butylene succinate). Fine Chem..

[44] Azim H., Dekhterman A., Jiang Z., Gross R.A. (2006). Candida antarctica lipase B-catalyzed synthesis of poly(butylene succinate): Shorter chain building blocks also work. Biomacromolecules.

[45] Debuissy T., Pollet E., Avérous L. (2016). Enzymatic Synthesis of a Bio-Based Copolyester from Poly(butylene succinate) and Poly((R)-3-hydroxybutyrate): Study of Reaction Parameters on the Transesterification Rate. Biomacromolecules.

[46] Ciulik C., Safari M., Martínez de Ilarduya A., Morales-Huerta J.C., Iturrospe A., Arbe A., Müller A.J., Muñoz-Guerra S. (2017). Poly(butylene succinate-ran-ε-caprolactone) copolyesters: Enzymatic synthesis and crystalline isodimorphic character. Eur. Polym. J..

[47] Safari M., Martínez De Ilarduya A., Mugica A., Zubitur M., Muñoz-Guerra S., Müller A.J. (2018). Tuning the Thermal Properties and Morphology of Isodimorphic Poly[(butylene succinate)- ran-(ε-caprolactone)] Copolyesters by Changing Composition, Molecular Weight, and Thermal History. Macromolecules.

[48] Pérez-Camargo R.A., Fernández-D’Arlas B., Cavallo D., Debuissy T., Pollet E., Avérous L., Müller A.J. (2017). Tailoring the structure, morphology, and crystallization of isodimorphic poly(butylene succinate-ran-butylene adipate) random copolymers by changing composition and thermal history. Macromolecules.

[49] Mincheva R., Delangre A., Raquez J.M., Narayan R., Dubois P. (2013). Biobased polyesters with composition-dependent thermomechanical properties: Synthesis and characterization of poly(butylene succinate-co-butylene azelate). Biomacromolecules.

[50] Díaz A., Franco L., Puiggalí J. (2014). Study on the crystallization of poly(butylene azelate-co-butylene succinate) copolymers. Thermochim. Acta.

[51] Pérez-Camargo R.A., Liu G., Cavallo D., Wang D., Müller A.J. (2020). Effect of the crystallization conditions on the exclusion/inclusion balance in biodegradable poly(butylene succinate-ran-butylene adipate) copolymers. Biomacromolecules.

[52] Tan B., Bi S., Emery K., Sobkowicz M.J. (2017). Bio-based poly(butylene succinate-co-hexamethylene succinate) copolyesters with tunable thermal and mechanical properties. Eur. Polym. J..

[53] Sisti L., Totaro G., Marchese P. (2016). PBS makes its entrance into the family of biobased plastics. Biodegradable and Biobased Polymers for Environmental and Biomedical Applications.

[54] Pérez-Camargo R.A., Arandia I., Safari M., Cavallo D., Lotti N., Soccio M., Müller A.J. (2018). Crystallization of isodimorphic aliphatic random copolyesters: Pseudo-eutectic behavior and double-crystalline materials. Eur. Polym. J..

[55] Pan P., Inoue Y. (2009). Polymorphism and isomorphism in biodegradable polyesters. Prog. Polym. Sci..

[56] Safari M., Mugica A., Zubitur M., Martínez De Ilarduya A., Muñoz-Guerra S., Müller A.J. (2020). Controlling the Isothermal Crystallization of Isodimorphic PBS-ran-PCL Random Copolymers by Varying Composition and Supercooling. Polymers.

[57] Arandia I., Mugica A., Zubitur M., Arbe A., Liu G., Wang D., Mincheva R., Dubois P., Müller A.J. (2015). How composition determines the properties of isodimorphic poly(butylene succinate-ran-butylene azelate) random biobased copolymers: From single to double crystalline random copolymers. Macromolecules.

[58] Arandia I., Mugica A., Zubitur M., Iturrospe A., Arbe A., Liu G., Wang D., Mincheva R., Dubois P., Müller A.J. (2016). Application of SSA thermal fractionation and X-ray diffraction to elucidate comonomer inclusion or exclusion from the crystalline phases in poly(butylene succinate-ran-butylene azelate) random copolymers. J. Polym. Sci. Part B Polym. Phys..

[59] Arandia I., Mugica A., Zubitur M., Mincheva R., Dubois P., Müller A.J., Alegría A. (2017). The Complex Amorphous Phase in Poly(butylene succinate-ran-butylene azelate) Isodimorphic Copolyesters. Macromolecules.

[60] Arandia I., Zaldua N., Maiz J., Pérez-Camargo R.A., Mugica A., Zubitur M., Mincheva R., Dubois P., Müller A.J. (2019). Tailoring the isothermal crystallization kinetics of isodimorphic poly (butylene succinate-ran-butylene azelate) random copolymers by changing composition. Polymer.

[61] Safari M., Leon Boigues L., Shi G., Maiz J., Liu G., Wang D., Mijangos C., Müller A.J. (2020). Effect of Nanoconfinement on the Isodimorphic Crystallization of Poly(butylene succinate- ran-caprolactone) Random Copolymers. Macromolecules.

[62] Yu Y., Wei Z., Liu Y., Hua Z., Leng X., Li Y. (2018). Effect of chain length of comonomeric diols on competition and miscibility of isodimorphism: A comparative study of poly(butylene glutarate-co-butylene azelate) and poly(octylene glutarate-co-octylene azelate). Eur. Polym. J..

[63] Yu Y., Wei Z., Zheng L., Jin C., Leng X., Li Y. (2018). Competition and miscibility of isodimorphism and their effects on band spherulites and mechanical properties of poly(butylene succinate-co-cis-butene succinate) unsaturated aliphatic copolyesters. Polymer.

[64] Yu Y., Wei Z., Zhou C., Zheng L., Leng X., Li Y. (2017). Miscibility and competition of cocrystallization behavior of poly(hexamethylene dicarboxylate)s aliphatic copolyesters: Effect of chain length of aliphatic diacids. Eur. Polym. J..

[65] Schäfer M., Yuan S., Petzold A., Pérez-Camargo R.A., Müller A.J., Thurn-Albrecht T., Saalwächter K., Schmidt-Rohr K. (2021). Asymmetric Co-unit Inclusion in Statistical Copolyesters. Macromolecules.

[66] Papageorgiou G.Z., Bikiaris D.N. (2007). Synthesis, cocrystallization, and enzymatic degradation of novel poly(butylene-co-propylene succinate) copolymers. Biomacromolecules.

[67] Debuissy T., Sangwan P., Pollet E., Avérous L. (2017). Study on the structure-properties relationship of biodegradable and biobased aliphatic copolyesters based on 1,3-propanediol, 1,4-butanediol, succinic and adipic acids. Polymer.

[68] Kluge M., Bikiaris D.N., Robert T. (2019). Enhancing the properties of poly(propylene succinate) by the incorporation of crystallizable symmetrical amido diols. Eur. Polym. J..

[69] Debuissy T., Pollet E., Avérous L. (2017). Synthesis and characterization of biobased poly(butylene succinate-ran-butylene adipate). Analysis of the composition-dependent physicochemical properties. Eur. Polym. J..

[70] Ye H.M., Wang R.D., Liu J., Xu J., Guo B.H. (2012). Isomorphism in poly(butylene succinate-co-butylene fumarate) and its application as polymeric nucleating agent for poly(butylene succinate). Macromolecules.

[71] Zheng L., Wang Z., Wu S., Li C., Zhang D., Xiao Y. (2013). Novel poly(butylene fumarate) and poly(butylene succinate) multiblock copolymers bearing reactive carbon-carbon double bonds: Synthesis, characterization, cocrystallization, and properties. Ind. Eng. Chem. Res..

[72] Wei X.W., Huang G., Wang J., Meng X., Zhou Q., Ye H.M. (2020). Tailoring crystallization of random terpolyester: Combination of isodimorphism and isomorphism. Macromolecules.

[73] Wei X.W., Yang L.L., Li Y., Meng X., Cai L.H., Zhou Q., Ye H.M. (2021). Asymmetrical formation of isomorphism in the crystalline/crystalline blend of poly(butylene succinate) and poly(butylene fumarate). Polymer.

[74] Poussard L., Mecheri A., Mariage J., Barakat I., Bonnaud L., Raquez J.M., Dubois P. (2014). Synthesis of oligo(butylene succinate)-based polyurethanes: Influence of the chemical structure on thermal and mechanical properties. J. Renew. Mater..

[75] Jamaluddin N., Razaina M.T., Ishak Z.M. (2016). Mechanical and Morphology Behaviours of Polybutylene (succinate)/Thermoplastic Polyurethaneblend. Procedia Chem..

[76] Kim T., Jeon H., Jegal J., Kim J.H., Yang H., Park J., Oh D.X., Hwang S.Y. (2018). Trans crystallization behavior and strong reinforcement effect of cellulose nanocrystals on reinforced poly(butylene succinate) nanocomposites. RSC Adv..

[77] Then Y.Y., Ibrahim N.A., Zainuddin N., Chieng B.W., Ariffin H., Zin Wan Yunus W.M. (2015). Effect of 3-aminopropyltrimethoxysilane on chemically modified oil palm mesocarp fiber/poly(butylene succinate) biocomposite. BioResources.

[78] Xu J., Manepalli P.H., Zhu L., Narayan-Sarathy S., Alavi S. (2019). Morphological, barrier and mechanical properties of films from poly(butylene succinate) reinforced with nanocrystalline cellulose and chitin whiskers using melt extrusion. J. Polym. Res..

[79] Petchwattana N., Covavisaruch S., Wibooranawong S., Naknaen P. (2016). Antimicrobial food packaging prepared from poly(butylene succinate) and zinc oxide. Meas. J. Int. Meas. Confed..

[80] Zainal Abidin A.S., Yusoh K., Jamari S.S., Abdullah A.H., Ismail Z. (2018). Surface functionalization of graphene oxide with octadecylamine for improved thermal and mechanical properties in polybutylene succinate nanocomposite. Polym. Bull..

[81] Ludueña L.N., Fortunati E., Morán J.I., Alvarez V.A., Cyras V.P., Puglia D., Manfredi L.B., Pracella M. (2016). Preparation and characterization of polybutylene-succinate/poly(ethylene-glycol)/cellulose nanocrystals ternary composites. J. Appl. Polym. Sci..

[82] Planellas M., Sacristán M., Rey L., Olmo C., Aymamí J., Casas M.T., Del Valle L.J., Franco L., Puiggalí J. (2014). Micro-molding with ultrasonic vibration energy: New method to disperse nanoclays in polymer matrices. Ultrason. Sonochem..

[83] Henke L., Zarrinbakhsh N., Endres H.J., Misra M., Mohanty A.K. (2016). Biodegradable and Bio-based Green Blends from Carbon Dioxide-Derived Bioplastic and Poly(Butylene Succinate). J. Polym. Environ..

[84] Phua Y.J., Chow W.S., Mohd Ishak Z.A. (2013). Reactive processing of maleic anhydride-grafted poly(butylene succinate) and the compatibilizing effect on poly(butylene succinate) nanocomposites. Express Polym. Lett..

[85] He L., Song F., Li D.F., Zhao X., Wang X.L., Wang Y.Z. (2020). Strong and Tough Polylactic Acid Based Composites Enabled by Simultaneous Reinforcement and Interfacial Compatibilization of Microfibrillated Cellulose. ACS Sustain. Chem. Eng..

[86] Dai X., Qiu Z. (2016). Synthesis and properties of novel biodegradable poly(butylene succinate-co-decamethylene succinate) copolyesters from renewable resources. Polym. Degrad. Stab..

[87] Jacquel N., Saint-Loup R., Pascault J.P., Rousseau A., Fenouillot F. (2015). Bio-based alternatives in the synthesis of aliphatic-aromatic polyesters dedicated to biodegradable film applications. Polymer.

[88] Genovese L., Lotti N., Gazzano M., Siracusa V., Dalla Rosa M., Munari A. (2016). Novel biodegradable aliphatic copolyesters based on poly(butylene succinate) containing thioether-linkages for sustainable food packaging applications. Polym. Degrad. Stab..

[89] Ojijo V., Sinha Ray S. (2013). Processing strategies in bionanocomposites. Prog. Polym. Sci..

[90] Ojijo V., Sinha Ray S. (2014). Nano-biocomposites based on synthetic aliphatic polyesters and nanoclay. Prog. Mater. Sci..

[91] Shirali H., Rafizadeh M., Afshar Taromi F., Jabbari E. (2017). Fabrication of in situ polymerized poly(butylene succinate-co-ethylene terephthalate)/hydroxyapatite nanocomposite to fibrous scaffolds for enhancement of osteogenesis. J. Biomed. Mater. Res.-Part A.

[92] Joy J., Jose C., Varanasi S.B., Mathew L.P., Thomas S., Pilla S. (2016). Preparation and characterization of poly(butylene succinate) bionanocomposites reinforced with cellulose nanofiber extracted from Helicteres isora plant. J. Renew. Mater..

[93] Saeed U., Nawaz M.A., Al-Turaif H.A. (2018). Wood flour reinforced biodegradable PBS/PLA composites. J. Compos. Mater..

[94] Chen Z., Gan L., Chang P.R., Liu C., Huang J., Gao S. (2017). Cost Reduction and Mechanical Enhancement of Biopolyesters Using an Agricultural Byproduct from Konjac Glucomannan Processing. ACS Sustain. Chem. Eng..

[95] Brody A.L. (2014). Packaging of Foods. Encycl. Food Microbiol. Second Ed..

[96] Li S.L., Wu F., Yang Y., Wang Y.Z., Zeng J.B. (2015). Synthesis, characterization and isothermal crystallization behavior of poly(butylene succinate)-b-poly(diethylene glycol succinate) multiblock copolymers. Polym. Adv. Technol..

[97] Robeson L.M. (2012). Polymer Membranes. Polymer Science: A Comprehensive Reference.

[98] Puchalski M., Szparaga G., Biela T., Gutowska A., Sztajnowski S., Krucińska I. (2018). Molecular and supramolecular changes in polybutylene succinate (PBS) and polybutylene succinate adipate (PBSA) copolymer during degradation in various environmental conditions. Polymers.

[99] Satti S.M., Shah A.A. (2020). Polyester-based biodegradable plastics: An approach towards sustainable development. Lett. Appl. Microbiol..

[100] Ayu R.S., Khalina A., Harmaen A.S., Zaman K., Mohd Nurrazi N., Isma T., Lee C.H. (2020). Effect of empty fruit brunch reinforcement in polybutylene-succinate/modified tapioca starch blend for agricultural mulch films. Sci. Rep..

[101] Lagarón J.-M. (2011). Multifunctional and Nanoreinforced Polymers for Food Packaging.

[102] Nazrin A., Sapuan S.M., Zuhri M.Y.M., Ilyas R.A., Syafiq R., Sherwani S.F.K. (2020). Nanocellulose Reinforced Thermoplastic Starch (TPS), Polylactic Acid (PLA), and Polybutylene Succinate (PBS) for Food Packaging Applications. Front. Chem..

[103] Siracusa V., Lotti N., Munari A., Dalla Rosa M. (2015). Poly(butylene succinate) and poly(butylene succinate-co-adipate) for food packaging applications: Gas barrier properties after stressed treatments. Polym. Degrad. Stab..

[104] Giacinti Baschetti M., Minelli M. (2020). Test methods for the characterization of gas and vapor permeability in polymers for food packaging application: A review. Polym. Test..

[105] Massey L.K. (2003). Permeability Properties of Plastics and Elastomers: A Guide to Packaging and Barrier Materials.

[106] Číhal P., Vopička O., Lanč M., Kludský M., Velas J., Hrdlička Z., Michalcová A., Dendisová M., Friess K. (2018). Poly(butylene succinate)-cellulose triacetate blends: Permeation, pervaporation, sorption and physical structure. Polym. Test..

[107] Guidotti G., Soccio M., Siracusa V., Gazzano M., Salatelli E., Munari A., Lotti N. (2017). Novel Random PBS-Based Copolymers Containing Aliphatic Side Chains for Sustainable Flexible Food Packaging. Polymers.

[108] Siracusa V., Rocculi P., Romani S., Rosa M.D. (2008). Biodegradable polymers for food packaging: A review. Trends Food Sci. Technol..

[109] Siracusa V., Blanco I., Romani S., Tylewicz U., Rocculi P., Rosa M.D. (2012). Poly(lactic acid)-modified films for food packaging application: Physical, mechanical, and barrier behavior. J. Appl. Polym. Sci..

[110] McHugh T.H., Krochta J.M. (1994). Sorbitol-vs Glycerol-Plasticized Whey Protein Edible Films: Integrated Oxygen Permeability and Tensile Property Evaluation. J. Agric. Food Chem..

[111] Rudnik E. (2019). Properties and Applications. Compostable Polymer Materials.

[112] Okamoto K., Ray S.S., Okamoto M. (2003). New poly(butylene succinate)/layered silicate nanocomposites. II. Effect of organically modified layered silicates on structure, properties, melt rheology, and biodegradability. J. Polym. Sci. Part B Polym. Phys..

[113] Charlon S., Follain N., Dargent E., Soulestin J., Sclavons M., Marais S. (2016). Poly[(butylene succinate)-co-(butylene adipate)]-Montmorillonite Nanocomposites Prepared by Water-Assisted Extrusion: Role of the Dispersion Level and of the Structure-Microstructure on the Enhanced Barrier Properties. J. Phys. Chem. C.

[114] Dean K.M., Pas S.J., Yu L., Ammala A., Hill A.J., Wu D.Y. (2009). Formation of Highly Oriented Biodegradable Polybutylene Succinate Adipate Nanocomposites: Effects of Cation Structures on Morphology, Free Volume, and Properties. J. Appl. Polym. Sci..

[115] Bhatia A., Gupta R.K., Bhattacharya S.N., Choi H.J. (2009). Effect of clay on thermal, mechanical and gas barrier properties of biodegradable poly(lactic acid)/poly(butylene succinate) (PLA/PBS) nanocomposites. Int. Polym. Process..

[116] Petersen K., Nielsen P.V., Olsen M.B. (2001). Physical and mechanical properties of biobased materials—Starch, polylactate and polyhydroxybutyrate. Starch/Staerke.

[117] Olabarrieta I., Forsström D., Gedde U.W., Hedenqvist M.S. (2001). Transport properties of chitosan and whey blended with poly(ε-caprolactone) assessed by standard permeability measurements and microcalorimetry. Polymer.

[118] Bastarrachea L., Dhawan S., Sablani S.S. (2011). Engineering Properties of Polymeric-Based Antimicrobial Films for Food Packaging. Food Eng. Rev..

[119] Xu Y., Zhang S., Wang P., Wang J. (2018). Synthesis of poly(butylene succinate) phosphorus-containing ionomers for versatile crystallization and improved thermal conductivity. Polymer.

[120] Cosquer R., Pruvost S., Gouanvé F. (2021). Improvement of barrier properties of biodegradable polybutylene succinate/graphene nanoplatelets nanocomposites prepared by melt process. Membranes.

[121] Xie L., Xu H., Chen J.B., Zhang Z.J., Hsiao B.S., Zhong G.J., Chen J., Li Z.M. (2015). From nanofibrillar to nanolaminar poly(butylene succinate): Paving the way to robust barrier and mechanical properties for full-biodegradable poly(lactic acid) films. ACS Appl. Mater. Interfaces.

[122] Messin T., Marais S., Follain N., Guinault A., Gaucher V., Delpouve N., Sollogoub C. (2020). Biodegradable PLA/PBS multinanolayer membrane with enhanced barrier performances. J. Memb. Sci..

[123] Messin T., Follain N., Guinault A., Sollogoub C., Gaucher V., Delpouve N., Marais S. (2017). Structure and Barrier Properties of Multinanolayered Biodegradable PLA/PBSA Films: Confinement Effect via Forced Assembly Coextrusion. ACS Appl. Mater. Interfaces.

[124] Duan R.T., He Q.X., Dong X., Li D.F., Wang X.L., Wang Y.Z. (2016). Renewable sugar-based diols with different rigid structure: Comparable investigation on improving poly(butylene succinate) performance. ACS Sustain. Chem. Eng..

[125] Attallah O.A., Mojicevic M., Lanzagorta Garcia E., Azeem M., Chen Y., Asmawi S., Fournet M.B. (2021). Macro and micro routes to high performance bioplastics: Bioplastic biodegradability and mechanical and barrier properties. Polymers.

[126] Risse S., Tighzert L., Berzin F., Vergnes B. (2014). Microstructure, rheological behavior, and properties of poly(lactic acid)/poly(butylene succinate)/organoclay nanocomposites. J. Appl. Polym. Sci..

[127] Salehiyan R., Ray S.S. (2018). Influence of Nanoclay Localization on Structure–Property Relationships of Polylactide-Based Biodegradable Blend Nanocomposites. Macromol. Mater. Eng..

[128] Ray S.S., Okamoto K., Okamoto M. (2003). Structure-property relationship in biodegradable poly(butylene succinate)/layered silicate nanocomposites. Macromolecules.

[129] Ilsouk M., Raihane M., Rhouta B., Meri R.M., Zicans J., Vecstaudža J., Lahcini M. (2020). The relationship of structure, thermal and water vapor permeability barrier properties of poly(butylene succinate)/organomodified beidellite clay bionanocomposites prepared by in situ polycondensation. RSC Adv..

[130] Saeng-on J., Aht-Ong D. (2018). Compatibility of banana starch nanocrystals/poly(butylene succinate) bio-nanocomposite packaging films. J. Appl. Polym. Sci..

[131] Preedanorawut R., Threepopnatkul P., Sittatrakul A. (2020). Effect of zeolite types on properties of polybutylene succinate/polylactic acid films. IOP Conf. Ser. Mater. Sci. Eng..

[132] Gain O., Espuche E., Pollet E., Alexandre M., Dubois P. (2005). Gas barrier properties of poly(ε-caprolactone)/clay nanocomposites: Influence of the morphology and polymer/clay interactions. J. Polym. Sci. Part B Polym. Phys..

[133] Follain N., Belbekhouche S., Bras J., Siqueira G., Chappey C., Marais S., Dufresne A. (2018). Tunable gas barrier properties of filled-PCL film by forming percolating cellulose network. Colloids Surfaces A Physicochem. Eng. Asp..

[134] Mao L., Wu H., Liu Y., Yao J., Bai Y. (2018). Enhanced mechanical and gas barrier properties of poly(ε-caprolactone) nanocomposites filled with tannic acid-Fe(III) functionalized high aspect ratio layered double hydroxides. Mater. Chem. Phys..

[135] Mohamed R.M., Yusoh K. (2015). A Review on the Recent Research of Polycaprolactone (PCL). Adv. Mater. Res..

[136] Kunioka M., Ninomiya F., Funabashi M. (2009). Biodegradation of poly(butylene succinate) powder in a controlled compost at 58 °C evaluated by naturally-occurring carbon 14 amounts in evolved CO_2_ based on the ISO 14855-2 method. Int. J. Mol. Sci..

[137] Anankaphong H., Pentrakoon D., Junkasem J. (2015). Effect of rubberwood content on biodegradability of poly(butylene succinate) biocomposites. Int. J. Polym. Sci..

[138] Ganesh Kumar A., Anjana K., Hinduja M., Sujitha K., Dharani G. (2020). Review on plastic wastes in marine environment—Biodegradation and biotechnological solutions. Mar. Pollut. Bull..

[139] Iram D., Riaz R.A., Iqbal R.K. (2019). Usage of potential micro-organisms for degradation of plastics. Open J. Environ. Biol..

[140] Brannigan R.P., Dove A.P. (2017). Synthesis, properties and biomedical applications of hydrolytically degradable materials based on aliphatic polyesters and polycarbonates. Biomater. Sci..

[141] Armentano I., Gigli M., Morena F., Argentati C., Torre L., Martino S. (2018). Recent advances in nanocomposites based on aliphatic polyesters: Design, synthesis, and applications in regenerative medicine. Appl. Sci..

[142] Gualandi C., Soccio M., Govoni M., Valente S., Lotti N., Munari A., Giordano E., Pasquinelli G., Focarete M.L. (2012). Poly(butylene/diethylene glycol succinate) multiblock copolyester as a candidate biomaterial for soft tissue engineering: Solid-state properties, degradability, and biocompatibility. J. Bioact. Compat. Polym..

[143] Huang A., Peng X., Geng L., Zhang L., Huang K., Chen B., Gu Z., Kuang T. (2018). Electrospun poly (butylene succinate)/cellulose nanocrystals bio-nanocomposite scaffolds for tissue engineering: Preparation, characterization and in vitro evaluation. Polym. Test..

[144] Zhang Y., Yuan W., Liu Y. (2018). Synthesis and characterization of bio-based poly(butylene succinate-co-10-hydroxydecanoate). J. Elastomers Plast..

[145] Sheikholeslami S.N., Rafizadeh M., Taromi F.A., Shirali H., Jabbari E. (2016). Material properties of degradable Poly(butylene succinate-co-fumarate) copolymer networks synthesized by polycondensation of pre-homopolyesters. Polymer.

[146] Morales-Huerta J.C., Ciulik C.B., De Ilarduya A.M., Muñoz-Guerra S. (2017). Fully bio-based aromatic-aliphatic copolyesters: Poly(butylene furandicarboxylate-co-succinate)s obtained by ring opening polymerization. Polym. Chem..

[147] Hu X., Gao Z., Wang Z., Su T., Yang L., Li P. (2016). Enzymatic degradation of poly(butylene succinate) by cutinase cloned from Fusarium solani. Polym. Degrad. Stab..

[148] Pan W., Bai Z., Su T., Wang Z. (2018). Enzymatic degradation of poly(butylene succinate) with different molecular weights by cutinase. Int. J. Biol. Macromol..

[149] Bai Z., Liu Y., Su T., Wang Z. (2018). Effect of hydroxyl monomers on the Enzymatic degradation of poly(ethylene succinate), poly(butylene succinate), and poly(hexylene succinate). Polymers.

[150] Shi K., Su T., Wang Z. (2019). Comparison of poly(butylene succinate) biodegradation by Fusarium solani cutinase and Candida antarctica lipase. Polym. Degrad. Stab..

[151] Li S.L., Wu F., Wang Y.Z., Zeng J.B. (2015). Biobased Thermoplastic Poly(ester urethane) Elastomers Consisting of Poly(butylene succinate) and Poly(propylene succinate). Ind. Eng. Chem. Res..

[152] Hwang S.Y., Jin X.Y., Yoo E.S., Im S.S. (2011). Synthesis, physical properties and enzymatic degradation of poly (oxyethylene-b-butylene succinate) ionomers. Polymer.

[153] Yang J., Tian W., Li Q., Li Y., Cao A. (2004). Novel biodegradable aliphatic poly(butylene succinate-co-cyclic carbonate)s bearing functionalizable carbonate building blocks: II. Enzymatic biodegradation and in vitro biocompatibility assay. Biomacromolecules.

[154] Huang X., Li C., Zheng L., Zhang D., Guan G., Xiao Y. (2009). Synthesis, characterization and properties of biodegradable poly(butylene succinate)-block-poly(propylene glycol)segmented copolyesters. Polym. Int..

[155] Han J., Shi J., Xie Z., Xu J., Guo B. (2019). Synthesis, properties of biodegradable poly(butylene succinate-co-butylene 2-methylsuccinate) and application for sustainable release. Materials.

[156] Kang Z.H., Wang C.L. (2013). Novel poly(butylenes succinate-block-1,3-propylene sebacate): Synthesis and enzymatic degradation. Adv. Mater. Res..

[157] Kong X., Qi H., Curtis J.M. (2014). Synthesis and characterization of high-molecular weight aliphatic polyesters from monomers derived from renewable resources. J. Appl. Polym. Sci..

[158] Wang L., Zhang M., Lawson T., Kanwal A., Miao Z. (2019). Poly(butylene succinate-co-salicylic acid) copolymers and their effect on promoting plant growth. R. Soc. Open Sci..

[159] Nikolic M.S., Djonlagic J. (2001). Synthesis and characterization of biodegradable poly(butylene succinate-co-butylene adipate)s. Polym. Degrad. Stab..

[160] Song D.K., Sung Y.K. (1995). Synthesis and characterization of biodegradable poly(1,4-butanediol succinate). J. Appl. Polym. Sci..

[161] Ferreira F.V., Dufresne A., Pinheiro I.F., Souza D.H.S., Gouveia R.F., Mei L.H.I., Lona L.M.F. (2018). How do cellulose nanocrystals affect the overall properties of biodegradable polymer nanocomposites: A comprehensive review. Eur. Polym. J..

[162] Scott G. (2000). “Green” polymers. Polym. Degrad. Stab..

[163] Nugroho P., Mitomo H., Yoshii F., Kume T., Nishimura K. (2001). Improvement of processability of PCL and PBS blend by irradiation and its biodegradability. Macromol. Mater. Eng..

[164] Haider T.P., Völker C., Kramm J., Landfester K., Wurm F.R. (2019). Plastics of the Future? The Impact of Biodegradable Polymers on the Environment and on Society. Angew. Chemie-Int. Ed..

[165] Kim H.S., Yang H.S., Kim H.J. (2005). Biodegradability and mechanical properties of agro-flour-filled polybutylene succinate biocomposites. J. Appl. Polym. Sci..

[166] Huang Z., Qian L., Yin Q., Yu N., Liu T., Tian D. (2018). Biodegradability studies of poly(butylene succinate) composites filled with sugarcane rind fiber. Polym. Test..

[167] Platnieks O., Gaidukovs S., Barkane A., Sereda A., Gaidukova G., Grase L., Thakur V.K., Filipova I., Fridrihsone V., Skute M. (2020). Bio-based poly(butylene succinate)/microcrystalline cellulose/nanofibrillated cellulose-based sustainable polymer composites: Thermo-mechanical and biodegradation studies. Polymers.

[168] Calabia B.P., Ninomiya F., Yagi H., Oishi A., Taguchi K., Kunioka M., Funabashi M. (2013). Biodegradable poly(butylene succinate) composites reinforced by cotton fiber with silane coupling agent. Polymers.

[169] Kim H.S., Kim H.J., Lee J.W., Choi I.G. (2006). Biodegradability of bio-flour filled biodegradable poly(butylene succinate) bio-composites in natural and compost soil. Polym. Degrad. Stab..

[170] Liu L., Yu J., Cheng L., Yang X. (2009). Biodegradability of poly(butylene succinate) (PBS) composite reinforced with jute fibre. Polym. Degrad. Stab..

[171] Teramoto N., Urata K., Ozawa K., Shibata M. (2004). Biodegradation of aliphatic polyester composites reinforced by abaca fiber. Polym. Degrad. Stab..

[172] Anstey A., Muniyasamy S., Reddy M.M., Misra M., Mohanty A. (2014). Processability and Biodegradability Evaluation of Composites from Poly(butylene succinate) (PBS) Bioplastic and Biofuel Co-products from Ontario. J. Polym. Environ..

[173] Phua Y.J., Lau N.S., Sudesh K., Chow W.S., Mohd Ishak Z.A. (2012). Biodegradability studies of poly(butylene succinate)/organo-montmorillonite nanocomposites under controlled compost soil conditions: Effects of clay loading and compatibiliser. Polym. Degrad. Stab..

[174] Platnieks O., Sereda A., Gaidukovs S., Thakur V.K., Barkane A., Gaidukova G., Filipova I., Ogurcovs A., Fridrihsone V. (2021). Adding value to poly(butylene succinate) and nanofibrillated cellulose-based sustainable nanocomposites by applying masterbatch process. Ind. Crops Prod..

[175] Platnieks O., Barkane A., Ijudina N., Gaidukova G., Thakur V.K., Gaidukovs S. (2020). Sustainable tetra pak recycled cellulose/poly(butylene succinate) based woody-like composites for a circular economy. J. Clean. Prod..

[176] Rojas-Lema S., Arevalo J., Gomez-Caturla J., Garcia-Garcia D., Torres-Giner S. (2021). Peroxide-induced synthesis of maleic anhydride-grafted poly(butylene succinate) and its compatibilizing effect on poly(butylene succinate)/pistachio shell flour composites. Molecules.

